# Release Is Not Dose: Speciation, Bioavailability and Cellular Signalling of Metal Ions from Orthodontic and Prosthetic Alloys in the Oral Environment

**DOI:** 10.3390/ijms27209318

**Published:** 2026-10-20

**Authors:** Marcin Mikulewicz

**Affiliations:** Department of Dentofacial Orthopaedics and Orthodontics, Division of Facial Abnormalities, Wroclaw Medical University, Krakowska 26, 50-425 Wroclaw, Poland; marcin.mikulewicz@umw.edu.pl; Tel.: +48-71-784-0299

**Keywords:** metal ion release, chemical speciation, bioaccessibility, nickel, orthodontic alloys, Toll-like receptor 4

## Abstract

Metal release from orthodontic and prosthetic alloys is measured almost universally as total elemental concentration in artificial or whole saliva and declared safe by comparison with dietary intake, drinking-water limits or toxic thresholds. This critical review argues that the comparison, a legitimate screen for systemic exposure, becomes a category error when used to exclude local mucosal effects. It traces the release literature’s reasoning since 1986 and shows that the extraction medium, not the alloy, determines the quantity and chemical form of what is measured. It then maps oral species onto cellular uptake routes and signalling pathways. Biological effect depends on species (aquo-ion, low-molecular-mass complex, protein adduct, oxyanion or particle); species dictate entry via divalent metal transporter 1, anion transporters, receptor-mediated endocytosis, membrane interaction or phagocytosis, thereby selecting between Toll-like receptor 4, NOD-like receptor family pyrin domain-containing 3, caspase-8, hypoxia-inducible factor 1α and non-apoptotic death programmes; and whole-saliva totals do not describe biofilm, crevicular or intracellular exposure. Oral speciation evidence rests on two nuclear magnetic resonance titrations and one chromium(VI) separation, and most mechanisms are borrowed from other tissues. A speciation-aware exposure model, presented as a hypothesis-generating framework, a minimum reporting set and prioritised experiments are proposed.

## 1. Introduction

Every fixed orthodontic appliance and every base-metal prosthesis corrodes. This is not a contested statement; it is the shared premise of four decades of measurement, and the measurements agree. Nickel, chromium, cobalt, molybdenum and titanium can be recovered from artificial saliva after the immersion of wires and brackets, from patient saliva during treatment and from the tissues that appliances touch. Clinically manifest reactions are nevertheless reported far less often than release is detected. A systematic review of hypersensitivity in orthodontics, restricted to subjects with at least one positive allergy test, assembled 858 subjects from 31 studies with 75 reported reactions, of which 29 were oral and 46 extra-oral or systemic, and with concomitant positive allergy tests recorded 451 times for nickel, 6 for cobalt, 5 for titanium and 4 for chromium [1]. Universal release and rare events sit side by side, and the field has reconciled them with a single sentence: the amount released is below dietary intake, therefore it is safe.

That sentence has a genealogy. Brune’s 1986 survey of metal release from dental biomaterials normalised corrosion-product quantities to a “standard restored man” and compared them with food and drink intake of the same elements as “one approach to a security analysis”, concluding that nickel from a high-nickel base-metal alloy could be released in vitro at the level of dietary intake, that mercury from amalgam and cadmium from copper amalgam approached it, and that release from the other alloys fell far below it [2]. Huang and colleagues, in the pH-series immersion study that became the template for two decades of archwire testing, reported that nickel released from four nickel–titanium (NiTi) wires at pH 2.5–6.25 was “well below the critical value necessary to induce allergy and below daily dietary intake level” [3]. A 2025 narrative review of nickel release from archwires concludes that short-term release is lower than harmful thresholds, modulated by pH, corrosion, treatment length and environment, while acknowledging that long-term studies are few and usually do not combine an in vitro and an in vivo arm [4]. The reassurance is inherited rather than re-derived, and that review’s admission that the two arms remain unbridged names the gap this article addresses.

The argument advanced here is that comparing a total elemental concentration with a dietary intake, while a legitimate conservative screen for systemic exposure, is a category error when it is used to exclude a local effect at the mucosa. A dietary limit is a statement about a systemic dose delivered through the gut to the whole body. A salivary or extract concentration is a statement about mass per volume in a fluid that is not the target tissue. What the mucosal epithelium, the gingival crevice and the resident immune cells encounter is neither a dose nor a concentration. It is a set of chemical species, each with its own ligand shell, oxidation state and physical form and, consequently, its own route into the cell and its own signalling consequence. Free aquo-nickel, nickel–histidine, nickel–lactate, nickel bound to albumin or mucin, chromium(III) hydroxo-species, chromium(VI) oxyanions, cobalt(II), titanium ions and titanium dioxide particles are different molecules, and a molecular journal is the right place to insist that they be treated as such. The relevant question for a Special Issue on the molecular and physicochemical study of orthodontic materials is not how much metal leaves the alloy but which molecules arrive at the receptor.

The same position has been reached independently outside dentistry. A critical appraisal of “systemic nickel allergy syndrome” argues that the entity fails the criteria for a discrete diagnosis partly because the supporting literature has largely neglected nickel bioavailability and bioaccessibility and because oral provocation tests use doses far exceeding dietary intake [5]. That is a single author’s argument rather than a dataset, and it is cited as corroboration of a way of reasoning rather than as evidence; but it is notable that an author writing in an allergy journal and a materials scientist arrive at the same fault line from opposite directions.

The review proceeds in seven steps. Section 2 characterises what the release literature measures and how it reasons, including the author’s own earlier use of the dietary comparison. Section 3 argues that in immersion studies the extraction medium is the experiment. Section 4 reviews the thin evidence on which species actually form in the mouth. Section 5 traces species to uptake routes and signalling pathways and assembles a speciation-aware exposure model. Section 6 reconciles clinical observations with bench results. Section 7 separates hazard from risk within the regulatory frame, and Section 8 proposes a minimum reporting set and a research agenda.

### Scope, Source Selection and Limits of the Evidence Base

This is a critical, argument-driven review, not a scoping or systematic review, and it does not claim the completeness that a Preferred Reporting Items for Systematic Reviews and Meta-Analyses extension for Scoping Reviews (PRISMA-ScR) search would provide. Sources were identified by the author through database (PubMed) and publisher searches on the release, speciation and cellular signalling of nickel, chromium, cobalt and titanium from dental alloys, supplemented by backward citation tracking from the included papers, and were selected purposively. For release studies, the papers that established the field’s reasoning (Brune 1986 [2]; Huang 2003 [3]) and representative recent in vitro and in vivo reports, including the author’s own [6,7]. For mechanisms, the original report of each uptake route or pathway rather than later confirmations. Finally, where available, primary studies from 2021–2026. No formal inclusion criteria, screening log or record counts were kept, and selective citation therefore cannot be excluded. This is why the framework of Section 4 and Section 5 is labelled hypothesis-generating, why the “typical design” column of the minimum-reporting-set table (Section 8) is tied to the specific studies it characterises rather than presented as a survey, and why the research agenda of Section 8 is a proposal. The 53 references comprise 38 published in 2020–2026 (20 in 2025–2026), 9 from 2010–2019 and 6 from before 2010, the earliest being Brune’s 1986 survey.

## 2. What the Release Literature Measures, and How It Reasons

### 2.1. Totals in Artificial Saliva

The in vitro literature shares one design. A component or set of components is immersed in a synthetic electrolyte at 37 °C for days to weeks, the supernatant is analysed by inductively coupled plasma mass spectrometry (ICP-MS), inductively coupled plasma optical emission spectrometry (ICP-OES) or atomic absorption spectrometry (AAS), and the concentration obtained is compared with an external limit. The author’s 2011 study is a fair specimen. Brackets, bands and wires immersed in artificial saliva yielded nickel at 573 µg/L against 15 µg/L in controls and chromium at 101 against 8 µg/L, with fourteen other elements also elevated. The results were then explicitly evaluated “in relation to the maximum acceptable concentrations of metal ions in drinking water and to recommended daily doses” and three release coefficients were defined to normalise them [6]. The framing was conventional, and it is the framing this review now rejects: Neither a drinking-water limit nor a daily dose describes what a gingival keratinocyte experiences. The author’s more recent comparison of stainless steel, NiTi, CrCo and titanium–molybdenum alloy (TMA) archwires after four weeks in artificial saliva showed by ICP-OES that NiTi and CrCo wires released significantly more nickel and chromium than stainless steel, and the discussion was again framed in terms of allergy risk and material selection [7]. The comparative ranking is sound. The step from ranking to biological consequence is not licensed by the data.

The same pattern recurs across the field. Uncoated, rhodium-coated and nitrided NiTi wires immersed for 28 days at pH 4.8–6.6 released titanium (all wires) and nickel (uncoated wires) in inverse proportion to pH while surface roughness was unaffected and hardness and modulus changed only marginally, and the authors observed that the concentrations were “below the recommended upper limit for daily intake; nevertheless, hypersensitivity effects cannot be excluded, even at lower concentrations and at low pH” [8]. That hedge is the honest response to a comparison the authors evidently did not find persuasive themselves. A component-resolved ICP-MS study found that release from stainless steel bands was an order of magnitude higher than from brackets, ligatures or NiTi wires, attributed to welding, that pitting was already present on as-received parts, that potassium chloride precipitated in the bath and that release did not correlate with roughness. Their paper opens by conceding that “despite numerous studies on ion release … no clear conclusions can be drawn due to complex interrelations of multiple factors” [9]. Huang’s pH series had already shown that manufacturer, pH and time all significantly affect release, with titanium mostly undetectable at pH ≥ 3.75, which the authors attributed to the protective TiO_2_ film [3], an observation that implied from the outset that what is measured is a property of the solution as much as of the alloy.

### 2.2. Compartments in the Patient

In vivo sampling is dominated by whole saliva. A random-effects meta-analysis of 14 observational studies found salivary nickel above baseline by 11.5 µg/L within ten minutes of appliance placement and by 1.38 µg/L at one day, with chromium raised by 6.25 µg/L at one day and 2.07 µg/L at one week. The pooled data thus show a transient early peak in the low-microgram-per-litre range, roughly 0.2 µM for nickel [10]. A three-month pilot study after debonding of 15 patients reported by ICP-OES that cobalt, chromium and titanium fell after removal, while nickel rose at three months, and concluded that “the levels were below the mean dietary intake levels; therefore, these concentrations can be considered non-toxic” [11]. The rise in nickel after removal is more interesting than the reassurance, which appears in the abstract while the body of the paper declines conclusions on safety. The rise in nickel is compatible with a reservoir that re-releases rather than a simple wash-out, although the authors list diet, pellicle or biofilm desorption and residues as alternatives and had no pre-treatment baseline.

Whole saliva is not, however, where the metal resides. A 12-month prospective cohort of 120 patients measuring nickel by graphite-furnace atomic absorption spectrometry (GF-AAS) in saliva, biofilm and cytobrush samples of buccal epithelium found progressive accumulation in biofilm from a median of 0.0008 to 21.58 µg/L (the concentration of a 0.5 mL suspension of a 3 mm^2^ scraping), biphasic kinetics in saliva and epithelial cells with a six-month peak, and by 9–12 months biofilm nickel approximately 1000-fold higher than the other compartments by the authors’ estimate, with treatment duration the strongest predictor and pH and age modulating biofilm accumulation [12]. The salivary medians in that cohort ranged from 0.002 to 19.5 µg/L across time points, from far below to above the meta-analytic increments cited above, which is itself a measure of how poorly whole-saliva totals agree between studies. Gingival crevicular fluid (GCF) does not resolve the disagreement; it illustrates one of its sources. In 24 patients followed for six months, GCF nickel measured by AAS rose roughly five-fold, to 19.8 µg/L, and chromium roughly seven-fold, to 13.8 µg/L, alongside a worsening gingival index [13]; baseline and six-month values are given in Table 1. The values were printed as µg/g and corrected to µg/L by a published erratum [13], a change of three orders of magnitude in meaning that the citing literature has largely not registered. In 60 patients sampled by ICP-MS, GCF nickel peaked at 101.78 µg/L at 30 days in the group using fluoridated oral hygiene agents and returned to baseline by six months, with chromium, titanium and manganese not significantly changed [14]. On the corrected units, crevicular nickel lies between the pooled whole-saliva increments of [10] and about ten times above them, not three orders of magnitude above as the uncorrected figures would imply. The compartment in contact with the junctional epithelium is therefore sampled rarely, reported in inconsistent units, and where the units are secure, not obviously richer in metal than saliva. The compartment that stands apart by orders of magnitude is the biofilm.

The metal also enters the cells. A PRISMA systematic review of 19 prospective and cross-sectional studies found higher metal content in oral epithelial cells during fixed-appliance therapy, accompanied by cytological and nuclear alterations and by cytotoxic and genotoxic effects that may depend on appliance composition and duration. These alterations tended to regress and did not progress to dysplasia [15]. That is a description of intracellular dose and its consequence, which is precisely what a salivary total is not.

Finally, the clinical endpoint does not track the total. In a prospective study of 30 adults sampled by ICP-MS at four time points, salivary ion concentrations did not differ between the end-of-treatment, start-of-treatment and control groups, yet the lymphocyte transformation test (LTT) detected nickel hypersensitivity in one-third of patients, including half of the start-of-treatment group, three of whom were newly sensitised after bracket insertion, and the most strongly sensitised individual had low salivary nickel. Borderline cases showed up to twenty times the reference value, and the authors concluded that reactions “occurred even with ion levels below thresholds” [16]. This is a null result for the correlation that the release literature assumes, and it should be read as such rather than explained away.

**Figure 1:**
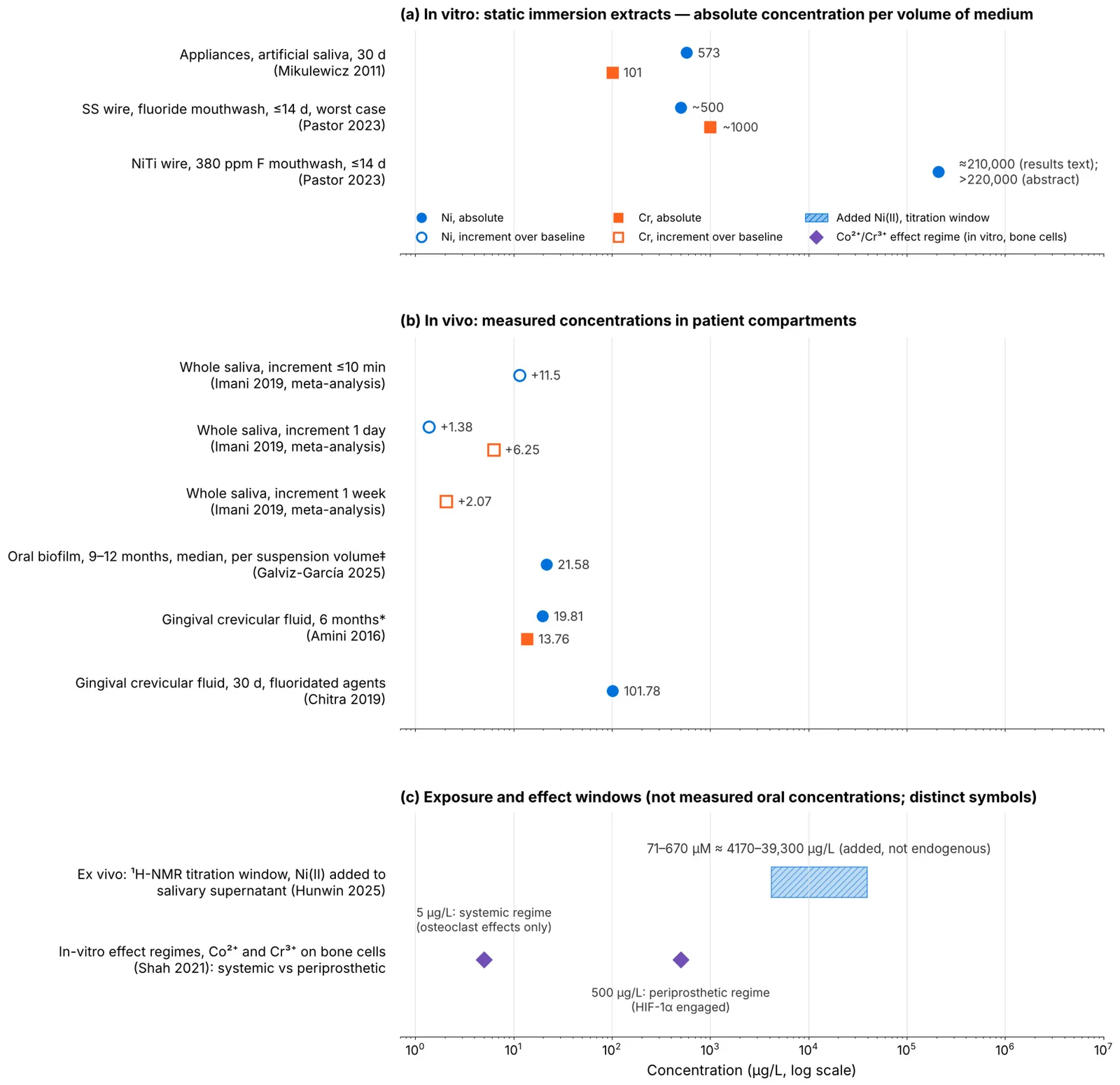
The dose gap. (**a**) In vitro immersion extracts: absolute concentrations in static baths, per volume of medium. (**b**) In vivo patient compartments: measured concentrations in whole saliva, biofilm and gingival crevicular fluid. (**c**) Exposure and effect windows, drawn with distinct symbols because they are not measured oral concentrations. The window in which Ni(II) was added to salivary supernatants for ^1^H NMR speciation (hatched band, added not endogenous) and the two concentration regimes of Co^2+^ and Cr^3+^ at which bone cells were profiled in [20] (diamonds; 5 µg/L systemic, 500 µg/L periprosthetic; note that these are not nickel). In (**a**,**b**), filled markers are absolute concentrations and open markers are increments above baseline. The biofilm value is the concentration of a 0.5 mL suspension of a 3 mm^2^ scraping, i.e., per suspension volume, not per mass of tissue. Nickel (circles) and chromium (squares) are plotted on a common log10 scale (µg/L). Reference values invoked by “below dietary intake” verdicts are not plotted because their units (µg/day, µg/kg/day, µg/cm^2^/week) have no counterpart on a concentration axis; they are tabulated in Supplementary Table S1. In vitro immersion extracts: Complete appliances in artificial saliva, Ni 573 µg/L and Cr 101 µg/L [6]; stainless steel wire in fluoride mouthwash at worst case, ~500 ppb Ni and ~1000 ppb Cr, and superelastic NiTi wire at 380 ppm fluoride, ≈210,000 ppb Ni in the results text of [17] (the abstract gives “exceeded 220,000 ppb”; both are shown, see Section 2.3) [17]; 1 ppb is taken as 1 µg/L. In vivo compartments: pooled mean differences from baseline in whole saliva, Ni +11.5 µg/L within ≤10 min and +1.38 µg/L at 1 day, Cr +6.25 µg/L at 1 day and +2.07 µg/L at 1 week (these are increments above baseline, not absolute concentrations) [10]; oral biofilm at 9–12 months, Ni 21.58 µg/L [12]; gingival crevicular fluid at 6 months, Ni 19.810 µg/L and Cr 13.760 µg/L [13] (* printed as µg/g in the original article and corrected to µg/L by a published erratum [13]); gingival crevicular fluid at 30 days under fluoridated oral hygiene, Ni 101.78 µg/L [14]. ‡ Speciation evidence: the concentration window in which the oral ^1^H-NMR speciation study of Ni(II) titrated it into salivary supernatants, 71–670 µmol/L [18], converted here to 4170–39,300 µg/L using M(Ni) = 58.69 g/mol. Studies differ in matrix, analytical method, sampling time and normalisation, so the chart compares orders of magnitude, not equivalent quantities.

Table 1 places the compartment concentrations reported in vivo against the concentrations obtained in immersion extracts and the concentration window of the only salivary speciation study (Section 4), converted to common molar units. Figure 1 plots the same data on a logarithmic axis. The gap between whole-saliva totals and both the interfacial compartments and the in vitro concentration spans several orders of magnitude, and in the current literature it is bridged by assumption alone.

**Table 1:** Reported total metal concentrations by oral compartment and in experimental systems, unit-harmonised. Values are absolute concentrations unless stated as increments over baseline. In vitro values are static immersion extracts (per volume of medium). The titration window is added, not endogenous, nickel. Values are totals by ICP-MS/OES, AAS or GF-AAS as published; harmonised values are computed here (µg/L ÷ molar mass = µM; Ni 58.69, Cr 52.00, Ti 47.87 g/mol) and rounded to three significant figures. Sources: [3,6,10,11,12,13,14,17,18,19].

Compartment/System	Element	Reported Value (As Published)	Harmonised Value (µg/L; µM)	Study Design/Sampling/*n* ^e^	Ref.
In vitro, artificial saliva (brackets, bands, wires)	Ni	573 µg/L (control 15 µg/L)	573 µg/L; 9.76 µM (control 0.256 µM)	Immersion 30 d, 36.6 °C, 25 mL; ICP-MS; *n* = 30 complete stainless steel appliance sets	[6]
In vitro, artificial saliva (brackets, bands, wires)	Cr	101 µg/L (control 8 µg/L)	101 µg/L; 1.94 µM (control 0.154 µM)	Immersion 30 d, 36.6 °C, 25 mL; ICP-MS; *n* = 30 complete stainless steel appliance sets	[6]
In vitro, artificial saliva pH 2.5–6.25, 1–28 d	Ni, Ti	Not tabulated here; “well below the critical value necessary to induce allergy and below daily dietary intake level”; Ti mostly undetectable at pH ≥ 3.75	not reported	Four NiTi wires, AAS	[3]
In vitro, fluoride mouthwash (130–380 ppm F), ≤14 d, stainless steel ^a^	Ni	~500 ppb (worst case; abstract and results figure); 50 µg/L in another passage of the text	~500 µg/L; ~8.52 µM	Immersion, ICP-MS; *n* = 5 wires per alloy per solution	[17]
In vitro, fluoride mouthwash (130–380 ppm F), ≤14 d, stainless steel ^a^	Cr	~1000 ppb (worst case; abstract and results figure); 71 µg/L in another passage of the text	~1000 µg/L; ~19.2 µM	Immersion, ICP-MS; *n* = 5 wires per alloy per solution	[17]
In vitro, 380 ppm F mouthwash, NiTi ^a^	Ni	≈210,000 ppb (results text); “exceeded 220,000 ppb” (abstract)	≈210,000 µg/L; ≈3580 µM (≈3.58 mM); abstract value > 220,000 µg/L, >3750 µM	Immersion, ICP-MS; *n* = 5 wires per alloy per solution	[17]
In vitro, 380 ppm F mouthwash, NiTi ^a^	Ti	≈180,000 ppb (results text); “exceeded 180,000 ppb” (abstract)	≈180,000 µg/L; ≈3760 µM (≈3.76 mM)	Immersion, ICP-MS; *n* = 5 wires per alloy per solution	[17]
In vitro, 380 ppm F mouthwash, Ti–Mo ^a^	Ti	≈140,000 ppb (results text); “reached 200,000 ppb” (abstract)	≈140,000 µg/L; ≈2920 µM (≈2.92 mM); abstract value ≈200,000 µg/L, ≈4180 µM	Immersion, ICP-MS; *n* = 5 wires per alloy per solution	[17]
In vivo, whole saliva (increment over baseline)	Ni	MD +11.5 µg/L at ≤10 min (95% CI 6.07–16.92); +1.38 µg/L at 1 day	+11.5 µg/L; 0.196 µM/+1.38 µg/L; 0.0235 µM	Meta-analysis, 14 observational studies	[10]
In vivo, whole saliva (increment over baseline)	Cr	MD +6.25 µg/L at 1 day; +2.07 µg/L at 1 week	+6.25 µg/L; 0.120 µM/+2.07 µg/L; 0.0398 µM	Meta-analysis, 14 observational studies	[10]
In vivo, oral biofilm, 0 → 9–12 months ^d^	Ni	Median 0.0008 → 21.58 µg/L; ~1000× saliva and buccal epithelial cells at 9–12 months (authors’ figure)	0.0008 µg/L; 0.0000136 µM → 21.58 µg/L; 0.368 µM	Prospective cohort, *n* = 120, GF-AAS	[12]
In vivo, gingival crevicular fluid, baseline → 6 months ^b^	Ni	3.894 ± 1.442 → 19.810 ± 8.452 µg/L	3.89 → 19.8 µg/L; 0.0663 → 0.338 µM	Prospective, *n* = 24, AAS	[13]
In vivo, gingival crevicular fluid, baseline → 6 months ^b^	Cr	1.978 ± 0.721 → 13.760 ± 3.555 µg/L	1.98 → 13.8 µg/L; 0.0380 → 0.265 µM	Prospective, *n* = 24, AAS	[13]
In vivo, gingival crevicular fluid, baseline → 30 d → 6 months (fluoridated-agent group)	Ni	Peak 101.78 µg/L at 30 d; returned to baseline at 6 months	101.78 µg/L; 1.73 µM	Prospective, *n* = 60 (two groups of 30), ICP-MS	[14]
Ex vivo, salivary supernatant, ^1^H NMR Ni(II) titration window ^c^	Ni (added)	71 µmol/L (low regime); 140–670 µmol/L (high regime)	≈4170 µg/L; 71 µM → ≈8220–39,300 µg/L; 140–670 µM	12 fasted volunteers, high-field ^1^H NMR	[18]
In vivo, whole saliva after appliance removal (0, 1, 3 months)	Co, Ni, Cr, Ti	Reported in µg/mL in the source; not tabulated here; “below the mean dietary intake levels”; Ni rose at 3 months (T3 vs T1, *p* = 0.029 as reported in the abstract; RM-ANOVA *p* = 0.006)	not reported	Pilot longitudinal, *n* = 15, ICP-OES	[11]
In vivo, intracellular (buccal epithelial cells), 3 months	Ni, Cr	Ni increase reported only in APF arm (*p* < 0.05); Cr not different from control; absolute values not tabulated here	not reported	RCT, *n* = 44, four arms, ICP-MS	[19]

^a^ ppb taken as µg/L, assuming an aqueous medium of density ≈ 1 g/mL; where the abstract and the results text of [17] give different figures, the results text value tied to the plotted data is used as the primary value and the abstract value is given alongside (tie-break rule and sensitivity in Section 2.3 and Supplementary Table S2). For stainless steel, the text also contains a passage giving 50 µg/L Ni and 71 µg/L Cr, a ten-fold discrepancy that the source does not resolve; the higher values are used because they match the abstract and the results figure, and the discrepancy is noted. ^b^ Printed as µg/g in the original article; a correction published by the authors states that all values should read µg/L [13]. The corrected unit is adopted here because it is the authors’ own correction and because the uncorrected unit would place crevicular metal three orders of magnitude above every other compartment, which no other GCF study supports (Section 2.2). ^c^ Added Ni(II) concentrations in a titration experiment, not endogenous salivary levels; µg/L = µmol/L × 58.69 [18]. ^d^ Medians; biofilm values are concentrations of a 0.5 mL phosphate-buffered saline suspension of a 3 mm^2^ biofilm scraping, and the “gingival” compartment of the source denotes buccal epithelial cells collected by cytobrush [12]. ^e^ Detection limits, recovery rates and sampling procedures are reported inconsistently across the sources and could not be tabulated uniformly; the column gives the sampling matrix, *n* and analytical method as published. This heterogeneity is itself a reporting gap addressed in Section 8. →, change from the first to the last time point stated. Abbreviations are defined in the list at the end of the article. “Not reported” indicates that the value is absent from the source publication.

### 2.3. Data Harmonisation

Four conversions underlie Table 1 and Figure 1, and each carries an assumption. (i) ppb → µg/L assumes an aqueous medium of density 1.00 g/mL. A mouthwash or artificial saliva of density 1.03 g/mL would change the values by 3%, immaterial at the orders-of-magnitude scale of the comparison. (ii) µg/g → µg/L for the crevicular data of [13] follows the authors’ published correction [13]; the two readings differ by a factor of 10^3^ and the choice is material, so both are tabulated in Supplementary Table S2 and the corrected unit is used for the reasons given in footnote b of Table 1. (iii) µmol/L → µg/L uses the atomic masses of Ni, Cr and Ti (58.69, 52.00, 47.87 g/mol) and carries no assumption beyond the identity of the element. (iv) Increments above baseline [10] are plotted as reported and are not absolute concentrations; they are distinguished by open markers in Figure 1. Where a source gives discordant values, the rule applied is that the value in the results section tied to the plotted or tabulated data is primary and the abstract value is reported alongside; for [17] this changes the NiTi nickel value from “>220,000” to ≈210,000 ppb and the Ti–Mo titanium value from ≈200,000 to ≈140,000 ppb (a factor of 1.05 and 1.43 respectively), and leaves a ten-fold internal discrepancy for stainless steel that the source does not resolve. None of these choices moves any point in Figure 1 across an order of magnitude except the µg/g–µg/L correction, which is the reason it is footnoted wherever it appears. The reference values that the “below dietary intake” verdicts invoke, with their units, are listed in Supplementary Table S1, and a sequential exposure calculation under stated assumptions is given in Supplementary Section S1.

## 3. The Extraction Medium Is the Experiment

### 3.1. pH and Fluoride

If release depends on the solution, the solution recipe is not a background condition but the principal independent variable. The pH dependence documented by Huang [3] and by Jusufi Osmani [8] is the mildest instance. A study of two molar-band brands aged for 48 h in artificial saliva at pH 4.5 or 7.4 followed by 500 thermocycles with Dulbecco’s modified Eagle medium (DMEM) extracts then tested on human gingival fibroblast-like cells by the MTT (3-(4,5-dimethylthiazol-2-yl)-2,5-diphenyltetrazolium bromide) viability assay and the comet assay found that one brand was significantly cytotoxic relative to control only after acidic ageing, with the longest comet tails at both pH values, while the other was non-cytotoxic at either pH, and that both produced measurable DNA damage at pH 4.5 [21]. Two nominally equivalent molar bands diverged, and the verdict on one of them changed with a single medium variable, although the direct pH 4.5 versus 7.4 and brand-to-brand comparisons did not reach significance. The endpoint was an extract of undefined composition, so the study establishes that the medium determines the answer without saying what the answer was in molecular terms.

Fluoride is the decisive case. In an ICP-MS study of stainless steel, Ti–Mo and superelastic NiTi wires immersed in mouthwashes at 130, 200 and 380 ppm fluoride for up to 14 days, stainless steel released at worst about 500 ppb nickel and 1000 ppb chromium, but at 380 ppm fluoride the Ti–Mo wires released about 200,000 ppb titanium and NiTi released more than 220,000 ppb nickel and 180,000 ppb titanium, with numerous pits on Ti–Mo, martensite plates in the NiTi austenitic matrix after four days and nickel-rich precipitates after more than seven days, with a loss of superelasticity at 37 °C [17]. That is not a scaling of release by a factor; it is a change in the chemistry of the interface by two to three orders of magnitude. The chemically expected cause is attack of the passive oxide by hydrofluoric acid with the formation of soluble titanium–fluoride complexes, a reaction that depends jointly on fluoride concentration and pH and proceeds once the hydrofluoric acid concentration exceeds roughly 30 ppm [22]. It was not measured in that study, and the pH of the mouthwashes is not reported. The exposure itself also departs from clinical use: a static immersion of up to 14 days has no counterpart in a rinse retained for one or two minutes a few times a day, so the worst-case figures describe the chemistry the alloy is capable of, not the dose a patient receives, and they carry a substantial risk of overestimation if read as exposure. Clinically used appliances behave differently again. Sets retrieved from 36 patients and new sets immersed in fluoride toothpaste, 1.23% acidulated phosphate fluoride (APF) or artificial saliva on a schedule scaled to three months of use and then extracted for seven days in DMEM, showed that used appliances released more ions, were rougher and were more cytotoxic to primary gingival fibroblasts than new ones, with APF increasing release and roughness and reducing viability most for TMA [23]. Prior intraoral exposure therefore conditions the alloy as well as the patient, and the DMEM that delivered the ions to the fibroblasts was a third medium, unrelated in composition to either the corrosion bath or saliva. The clinical counterpart is a randomised trial of 44 patients in four arms in which intracellular nickel in buccal cells, measured by ICP-MS, rose significantly only in the acidulated-phosphate-fluoride arm. No arm differed from control in chromium change, and APF reduced cell viability and increased morphological death markers without changing micronucleus frequency [19]. A chemical modifier of speciation changed uptake and viability but not the genotoxic endpoint, which is the dissociation between total, delivered and effective dose that this review is about.

### 3.2. Proteins, Buffers and Replenishment

Artificial saliva as usually formulated contains no protein [24], and the omission is not neutral. Immersion of a complete fixed appliance in a proteinaceous yeast-peptone-dextrose (YPD) medium versus artificial saliva under two replenishment regimes, analysed by ICP-MS, atomic force microscopy (AFM), scanning electron microscopy with energy-dispersive X-ray spectroscopy (SEM/EDX) and X-ray photoelectron spectroscopy (XPS), showed that the protein-containing medium promoted leaching of iron, copper and aluminium while suppressing nickel and chromium. Nickel and chromium species were detected in the top surface layer of NiTi in artificial saliva but not in the protein medium; and switching from static to renewed medium altered both the surface chemistry and leaching [25]. The direction of the protein effect is element- and alloy-specific, and it reverses for cobalt–chromium–molybdenum (CoCrMo) alloy. At pH 7.3, 10 g/L bovine serum albumin (BSA) and 2.5 g/L fibrinogen strongly enhanced the release of cobalt, chromium and molybdenum from wrought and laser-powder-bed-fusion CoCrMo while lowering friction, wear-track width and tribocorrosion, and it abolished the local acidification and anodic–cathodic separation manifest as strong molybdenum oxidation in the protein-free wear track. The authors had to choose a 2-(N-morpholino)ethanesulfonic acid (MES) rather than a phosphate buffer to prevent metal–phosphate and metal–protein aggregate precipitation [26]. That last detail deserves emphasis. The buffer selected to hold the pH determines whether the released metal remains in solution to be measured at all, which means that a phosphate-buffered artificial saliva can under-report release by precipitating it. Proteins simultaneously change how much is released and what form it takes, and a medium without them measures a different reaction.

### 3.3. Manufacturing Route, Recipe Heterogeneity and Irreproducibility

The material side is no more stable. Selective-laser-melted Co–Cr disks fabricated from recycled powder released more cobalt and chromium into DMEM over 24 h and 7 days and were more cytotoxic than disks from virgin powder. The same study documented concentration-dependent apoptosis, reactive oxygen species (ROS), and increased levels of hypoxia-inducible factor 1α (HIF-1α), tumour necrosis factor α (TNF-α), interleukin (IL)-6, IL-8 and vascular endothelial growth factor (VEGF) in gingival fibroblasts and oral keratinocytes [27]. A practical guide to simulated body fluids for corrosion testing dental-implant alloys compiles the artificial-saliva and simulated-body-fluid recipes in use, standardised to g/L. The guide also maps pH, halide, calcium–phosphate balance, protein, gas control and flow to specific corrosion modes, identifies the lack of a standardised fluoride–pH matrix and the limited guidance for microbiome-integrated assays, and warns that “quantitative links between simulated-fluid testing and clinical outcomes … remain insufficiently established” [24]. There is, in other words, no single artificial saliva, and no recipe has been validated against clinical exposure.

Standardisation does not rescue the measurement. In a United States Food and Drug Administration (FDA)-led inter-laboratory study, eight laboratories performed International Organization for Standardization (ISO) 10993-18 [28] extraction of two polymers, each compounded with nine known chemicals, in two solvents. The central 90% ranges of repeatability and reproducibility relative standard deviation (RSD) were 0.09–0.22 and 0.30–0.85, and the reproducibility limit implied that two laboratories using the same extraction conditions could report results differing by up to 240% (95% confidence, 95% of systems), with the analytical method alone a substantial contributor [29]. If the regulated extract of a compounded polymer can differ by up to 240% between competent laboratories using the same conditions, unregulated artificial-saliva immersions of corroding alloys cannot be expected to do better. An adjacent regulated field has drawn the conclusion. A validated ICP-MS and cold-vapour atomic fluorescence spectrometry workflow comparing total metal content in tattoo inks after acid digestion with the bioaccessible fraction in deionised water, artificial sweat and physiological saline found that bioaccessibility depended strongly and significantly on the medium, nickel reaching 63% solubility in saline and 59% in artificial sweat in black inks and mercury up to 72% in white inks, while legislation limits soluble fractions without specifying the medium [30]. The bioaccessible fraction is a property of the material–medium pair, not of the material. Table 2 summarises the medium variables that the studies above have shown to change the release result and, more importantly, the released species.

## 4. Which Species Actually Form in the Mouth (A Hypothesis-Generating Framework)

### 4.1. Two Titrations and One Separation

The honest starting point is that the speciation of alloy-derived metals in human saliva has been examined directly by one group, in two proton nuclear magnetic resonance (^1^H NMR) titrations: cobalt(II) in 2012 [31] and nickel(II) in 2025 [18]. A high-field ^1^H NMR titration of nickel(II) into whole-mouth salivary supernatants from twelve fasted volunteers showed that at low added nickel (71 µmol/L) histidine, taurine and methylamines, nitrogen-donor ligands with high stability constants, are the dominant complexants, whereas at 140–670 µmol/L the far more abundant carboxylate oxygen-donors lactate, formate, succinate and acetate take over. Ethylenediaminetetraacetic acid (EDTA) largely reversed the resonance broadenings (some regenerated signals retained partial broadening), consistent with transferable low-molecular-mass complexes, and the authors explicitly linked the identity of these complexes to the corrosion of nickel-containing dental alloys and to the carcinogenic potential of low-molecular-mass nickel(II) species [18]. The earlier cobalt(II) titration in the same design found lactate > formate ≈ histidinate > succinate as the most responsive complexants, an order set jointly by stability constants and salivary concentrations, and noted that these ligands are also hydroxyl-radical scavengers, so that any radical generated from complexed Co(II) by pseudo-Fenton chemistry may be scavenged at its site of formation [31]. Three consequences follow. Salivary nickel is not predominantly an aquo-ion, and the free-aquo fraction was not quantified. Its ligand shell changes with concentration, so that the species present at the sub-micromolar whole-saliva level and at the hundreds-of-micromolar crevicular level are different molecules with different charge, size and exchange kinetics. Furthermore, the complexes are labile, which means that the species arriving at a membrane receptor is decided by ligand competition at that membrane, not by the bulk composition of saliva. Everything else in this section is inference from other compartments, and it is presented as such. Two further limits of these titrations should be stated. The metal was added as a salt to fasted supernatant, so the ligand hierarchy describes clean saliva rather than the crevice of a patient with an appliance, where mucin, histidine-rich histatins and bacterial metabolites change the ligand pool. The lowest titration point, 71 µmol/L, lies between two and three orders of magnitude above the whole-saliva increments of Section 2, so that the species distribution at the concentrations most often reported has not been observed at all, only extrapolated from the high-affinity end of the titration. In concrete terms, 71 µmol/L (≈4170 µg/L) is about 41 times the highest crevicular nickel concentration reported (101.78 µg/L, 1.73 µM [14]) and about 360 times the pooled whole-saliva increment (11.5 µg/L, 0.196 µM [10]). The titration results are therefore not extrapolated to the patient range here. The species identified in these titrations are those observed at the titration concentrations, not species demonstrated in patients. Two further caveats apply. ^1^H NMR lacks the sensitivity to resolve species at low-micromolar concentrations and cannot serve as a routine speciation tool for patient saliva, and neither the cobalt(II) titration (2012) nor the nickel(II) titration (2025) has been validated in subjects wearing orthodontic appliances.

### 4.2. Modelling, Proteins and the Corona

Thermodynamic speciation modelling is a tool that, to the author’s knowledge, the dental release literature has not applied to alloy-derived nickel, chromium or cobalt in saliva. Applied to the aluminium and chromium salts used in patch testing, modelling with JESS, Hydra/Medusa and Visual MINTEQ showed that bioavailable fraction and chemical form depend strongly on salt type, applied concentration, sweat composition and pH, and that the salts themselves shift pH, dichromate raising it and aluminium and chromium(III) chlorides strongly lowering it, which the authors suggest may increase skin diffusion. They argue that this likely explains the reported seasonal and interpatient variability in aluminium patch-test positivity [32]. The same approach applied to artificial and real saliva, with the ligand inventory that the NMR study supplies, would convert a total concentration into a species distribution, and it is the first item on the agenda in Section 8.

Once protein is present, the metal–protein adduct is not an inert carrier but a new chemical entity. Cobalt(II) and chromium(III), delivered as salts pre-selected by speciation modelling so as neither to shift pH nor to precipitate at 150 mM NaCl and pH 7.3, induced or accelerated aggregation of bovine serum albumin, individually and as an equimolar mixture, as shown by spectroscopy, light scattering and melting curves [33]. An aggregated albumin is a different ligand for endocytic receptors from the native protein. In the mouth, the relevant protein pool is the pellicle. Liquid chromatography–tandem mass spectrometry of pellicles formed in vitro from pooled saliva on polished grade 2 titanium and feldspathic ceramic (implant-abutment materials) identified 396 adsorbed proteins out of 759 in pooled saliva, 369 on titanium and 298 on ceramic with 281 in common, and gene-ontology annotation of immune processes identified fourteen hub proteins linking multiple immune processes among the adsorbed proteins [34]. No metal surface in the mouth contacts tissue bare, and the corona through which every ion must pass differs between materials and contains a network of the proteins the immune system reads.

### 4.3. Oxidation State, Fluoride and Compartments

For chromium, the decisive speciation variable is oxidation state. Chromium leaching from a solid mineral matrix at pH 7.4 emerged specifically in the hexavalent, genotoxic state upon hydrogen peroxide oxidation. Epithelial, mesothelial and carcinoma cell lines expressing membrane-bound chromium(VI) transporters accumulated chromium up to ten-fold relative to the medium concentration, and anion-transporter inhibitors reduced uptake up to 45-fold [35]. The mineral was chrysotile, not an alloy, but the chemistry transfers. A passive chromium oxide sheds chromium(III). Whether an inflamed crevice can oxidise it to chromium(VI) is untested. The reaction requires a strong oxidant, and it has not been determined whether the hydrogen peroxide, myeloperoxidase and microbial metabolites of oral inflammation reach sufficient concentrations. The oxidation in [35] was catalysed by a mineral surface. Physiological peroxide concentrations are far lower, and chromium(III) in vivo is rapidly bound by transferrin and small carboxylates; only the hexavalent oxyanion has an anion-transporter route into the cell. This is the most important untested premise of the chromium argument and is placed on the agenda in Section 8; chromium(VI) has not been demonstrated in the gingival crevice. A total chromium measurement cannot distinguish the two, and none of the release studies cited in Section 2 attempted to. One study outside that set stated that after 24 h immersion of stainless steel brackets in synthetic saliva at pH 6.0 and 37 °C, separation on aminopropyl-functionalised silica followed by GF-AAS recovered about 40% of the total chromium released as chromium(VI) [36]. The method was validated in the source. It included separation on 150 mg of (3-aminopropyl)triethoxysilane-functionalised silica at pH 8 after a one-minute contact, quantification by GF-AAS with calibration against Cr(VI) standards for the hexavalent fraction and against stoichiometric Cr(III) + Cr(VI) mixtures for total chromium (the two species gave different sensitivities), a detection limit of 0.1 µg/L for both total and hexavalent chromium, spike recoveries of 97–110% in synthetic saliva, inter-day repeatability below 6% RSD, no Cr(III)⇌Cr(VI) interconversion in spiked mixtures held for 1 h at 37 °C, no oxidation of metallic chromium powder processed in the same way, and no effect of 0.1% mucin on the Cr(VI) result. Neither alkaline digestion nor diphenylcarbazide colorimetry was used, so the two classical sources of artefactual oxidation do not apply. The limits are of scope rather than of method: one protein-free synthetic saliva recipe (phosphate–citrate–lactate–thiocyanate–urea, pH ≈ 6), a single 24-h agitated immersion, 20 stainless steel brackets of two models selected for high corrosion, an interconversion test run for 1 h in the absence of the alloy rather than over the 24-h corrosion period in its presence, and no repetition by another laboratory or in crevicular fluid or biofilm. The 40% figure, therefore, serves here as existence evidence that Cr(VI) can leave a corroding stainless steel bracket in a saliva-like electrolyte, not as a quantitative input to any exposure estimate, and it is used in that sense only. A minimal verification would run the same recipe and bracket model in parallel with and without an oxidation inhibitor and in a protein-containing medium before the finding is extended beyond the bench. If it holds in saliva, the hexavalent species need not await oxidation in the crevice. It can leave the alloy in that state, which makes the speciation of released chromium, rather than its total, the measurement on which the entire chromium argument turns.

Fluoride, which has already been discussed, belongs here because its effect on titanium is a change of species rather than of quantity. Titanium leaves NiTi and Ti–Mo wires at 380 ppm fluoride in quantities two to three orders of magnitude above those from steel [17]. That it does so as fluoro-complexes formed by dissolution of the passive layer is the chemically expected route but was not tested in that study. Whether complexes, hydrolysed ions or oxide particles reach the epithelium is unknown. The compartment data of Section 2 complete the picture. Biofilm holds nickel at roughly 1000 times the salivary and epithelial-cell concentration after 9–12 months of treatment, by the authors’ estimate [12], and GCF carries nickel and chromium at 14–20 µg/L after six months [13], with a transient nickel peak near 100 µg/L under fluoride exposure [14], the same order as whole saliva. Neither compartment has been speciated. The biofilm fraction is presumably bound to extracellular polymer and bacterial surfaces and behaves as a reservoir, which would be consistent with the post-debonding nickel rise [11], but “presumably” is the operative word. The evidentiary base for Section 5 is therefore two NMR titrations of added cobalt and nickel in saliva, one chromium(VI) separation from a bracket extract, a speciation model developed for sweat, protein chemistry from serum and chromium redox chemistry from a mineral. It is enough to define the question and not enough to answer it.

## 5. From Species to Cell: Uptake Routes and Signalling (A Hypothesis-Generating Framework)

### 5.1. Four Routes In

Uptake is where speciation becomes dose. The divalent nickel ion enters cells through the divalent metal transporter 1 (DMT1). A comparison of human embryonic kidney (HEK) cells overexpressing DMT1 with A549 cells expressing only endogenous transporters showed that nickel(II) enters via DMT1 in competition with iron(II). The comparison also showed that soluble nickel lowered total cellular iron by about 40%, inactivated cytosolic aconitase and converted it to iron-regulatory protein 1 (IRP1), raised transferrin-receptor mRNA and produced a pseudo-hypoxic state with HIF-1α stabilisation at normal oxygen tension [37]. That single route already couples uptake to signalling. Nickel impairs the iron- and ascorbate-dependent prolyl hydroxylases that mark HIF-1α for degradation, whether by lowering the cellular iron pool [37] or, as a study that found no change in cellular iron proposed, by depleting intracellular ascorbate [38]; the relative weight of the two is unresolved. A comprehensive review of cobalt, nickel and manganese toxicology treats their exposure, homeostasis and transport together and adds a route the dental literature ignores, namely direct interaction of the metals with membrane lipids, producing rigidification, leakage that alters membrane potential, lipid phase changes, altered lipid metabolism and morphological change [39]. Membrane interaction requires no transporter and is available to the aquo-ion and to labile complexes alike.

Protein-bound metal takes a third route, and the cleanest demonstration that the carrier rather than the quantity sets the dose comes from the kidney. In a rat proximal tubule cell line, cadmium complexed with β2-microglobulin, albumin or lipocalin-2 at physiological ultrafiltrate concentrations was endocytosed via megalin:cubilin and was toxic, whereas the apo-proteins were not. Megalin:cubilin-deficient cells were protected, and cadmium–metallothionein was non-toxic even at more than 100-fold physiological concentration [40]. Which protein carries the metal, not how much metal is present, determined uptake and toxicity, and there is no reason to expect the albumin- and mucin-rich crevice to behave otherwise. The fourth route is anion transport of chromium(VI), with its up-to-tenfold accumulation and 45-fold inhibitor sensitivity [35]. Particulate forms add phagocytosis, discussed below.

### 5.2. The Receptor Sees a Ligand, Not an Element

The molecular anchor of the entire argument is the finding that nickel(II) directly activates human Toll-like receptor 4 (TLR4) as a non-protein, non-lipopolysaccharide (LPS) agonist, generating a proinflammatory signal independent of the T-cell signal. Activation is absent in mouse TLR4. It requires the non-conserved human histidines His456 and His458 (which are dispensable for LPS), and transgenic human TLR4 restored nickel sensitisation and elicitation of contact hypersensitivity in *Tlr4*-deficient mice [41]. The two non-conserved histidines are proposed to coordinate the ion through their imidazole nitrogens, although no structure of the complex has been determined. This is coordination chemistry, and it imposes a condition that no concentration measurement captures. Nickel already held by histidine or methylamine ligands in saliva, or by histidine-rich proteins in the pellicle, must exchange ligands to bind the receptor, and whether it does so depends on the relative stability constants and exchange kinetics of the competing complexes. A nickel–histidine complex is a different ligand for TLR4 from an aquo-ion, and at the hundreds-of-micromolar concentrations at which carboxylates dominate in the salivary titration [18], which no oral compartment measured to date reaches, the competing species are different again, so that the ligand hierarchy at the sub-micromolar concentrations of saliva and crevicular fluid has not been observed. The follow-up single-cell study from the same group shows where the response begins. Within eight hours of epicutaneous nickel challenge, endothelial cells, suprabasal keratinocytes, fibroblasts and C-C chemokine receptor type 7-positive (CCR7^+^) dendritic cells co-expressing the TLR4-interacting proteoglycan decorin and C-C motif chemokine ligand 21 (CCL21) were transcriptionally activated. Skin-resident T cells were unchanged, with massive Krüppel-like factor 2-positive (KLF2^+^) central-memory T-cell infiltration only at 72 h [42]. The first responders are barrier and stromal cells reading an innate receptor at the interface. The compartment in which speciation matters is therefore the epithelium and its immediate fluid, not the systemic circulation that a dietary comparison implicitly addresses.

### 5.3. Same Element, Different Pathways

The experimental proof that total metal cannot predict effect comes from macrophages exposed to CoCrMo corrosion products. In LPS-primed bone-marrow-derived macrophages from wild-type, *Nlrp3*^−/−^ and *Casp1*^−/−^ mice, IL-1β release induced by 60-nm Cr_2_O_3_ particles and by Cr^3+^ was dependent on NOD-like receptor family pyrin domain-containing 3 (NLRP3) and caspase-1, the particles acting through cathepsin B and lysosomal destabilisation and the ion through ROS, whereas IL-1β release induced by 3.4-µm CoCrMo particles and by Co^2+^ was NLRP3-independent and caspase-8-dependent (caspase-1-independent for the particles and only partly caspase-1-dependent for Co^2+^) [43]. Four physicochemical forms of the same two elements engaged four mechanistically distinct routes to the same cytokine in the same cell. An ICP-MS total of cobalt and chromium in the supernatant would have been blind to a difference that was decisive at the level of the inflammasome.

Particulate nickel is a separate case again. Intratracheal nano-nickel at 50 µg per mouse activated NLRP3 with IL-1β production and extensive fibrosis in wild-type mice but produced only mild changes in *Nlrp3*^−/−^ or *Il1r*^−/−^ animals. In MH-S alveolar macrophages, it acted strictly as a second signal, activating the inflammasome only in LPS-primed cells and doing so through upregulation of histone deacetylase 6 (HDAC6), validated by a small interfering RNA knockdown [44]. Ionic nickel is a TLR4 agonist; particulate nickel is an NLRP3 second signal that does nothing in an unprimed cell. The priming requirement introduces a conditionality that no total-release comparison can represent: the same delivered dose is inert in a healthy mucosa and active in one that carries a bacterial pattern. Titanium provides the oral tissue demonstration. Titanium ions enhanced differentiation of monocytic myeloid-derived suppressor cells (M-MDSCs) and macrophages from marrow precursors, acted synergistically with LPS to expand the suppressor population, and in a ligature-induced peri-implantitis model the peri-implant suppressor cells expanded, upregulated the osteoclastogenic receptor c-Fms accompanied by falls in peri-implant bone mineral density and bone volume [45]. That study tested ions only; that titanium ion and titanium dioxide particle are not interchangeable exposures follows from the particle/ion dissociation shown for cobalt and chromium [43], and the ion’s effect is conditional on a microbial context that no extract assay reproduces. The mouth is the one body compartment in which that context is never absent. An appliance sits in biofilm from the day it is placed, and the crevice adjacent to a band is by definition a site of bacterial pattern exposure. A cell model that omits the priming signal is not a conservative simplification of the oral interface. It is a model of a different tissue, and the null results it produces cannot be transferred to the primed state. Particulate exposure completes the set of physical forms. Wear and corrosion debris, the oxide fragments shed from a fluoride-attacked passive layer and the nanoparticles that form when released ions precipitate with phosphate are handled by phagocytosis and lysosomal processing rather than by any transporter, and the inflammasome studies above show that the physical form of the debris, 60-nm Cr_2_O_3_ against 3.4-µm CoCrMo particles, which differ in composition as well as size, selects the pathway [43]. None of these forms is distinguished by acid digestion and ICP-MS, which return the same number for a particle, a complex and an ion.

For chromium, the pathway repertoire attributed to “chromium” in the release literature belongs almost wholly to the hexavalent species. Chromium(VI) drives receptor-interacting protein kinase (RIPK) 1/RIPK3 necroptosis, NLRP3/caspase-1/gasdermin D (GSDMD) pyroptosis with IL-1β and IL-18 secretion, and ferroptosis through redox imbalance and iron accumulation, with ROS positioned as the rheostat that selects among them [46]. It is reasonable to read these as consequences of intracellular chromium(VI) reduction, and on that reading they are not available to the chromium(III) that a passive alloy sheds unless the crevice oxidises it first, although chromium(III) itself drives ROS-dependent NLRP3/caspase-1 IL-1β release in primed macrophages [43]. Cobalt(II) also stabilises HIF-1α through mechanisms that include substitution for iron at the prolyl-hydroxylase active site, depletion of the cofactor ascorbate [38] and direct binding to the degradation domain of HIF-α that blocks its recognition by pVHL [47]; the relative contribution of each is unresolved. The release-to-signalling bridge in oral cells is the study in which cobalt- and chromium-containing DMEM produced concentration-dependent apoptosis, ROS and increased HIF-1α, TNF-α, IL-6, IL-8 and VEGF levels in gingival fibroblasts and keratinocytes [27]. The medium was DMEM, so the species were those of a bicarbonate-buffered, amino-acid-rich culture medium rather than of saliva; but it is one of very few studies in which release and a defined pathway were measured together.

### 5.4. Concentration Regime as a Categorical Variable

The most instructive non-dental experiment compares primary human osteoblasts, osteoclast precursors and mature osteoclasts exposed to cobalt(II), chromium(III) and CoCr nanoparticles at systemic-circulating versus local periprosthetic concentrations by whole-genome microarray. At systemic levels, osteoblasts were unaffected and only osteoclast differentiation and function were inhibited through focal-adhesion and matrix-interaction pathways. At periprosthetic levels, both lineages were inhibited, via HIF-1α signalling in osteoblasts and endocytic and cytoskeletal genes in osteoclasts, with added inflammatory signalling and gene clusters and Kyoto Encyclopedia of Genes and Genomes (KEGG) pathways whose expression correlated with increasing Co^2+^ and Cr^3+^ concentrations [20]. Concentration regime here is not a position along one dose–response curve but a switch between transcriptional programmes. The systemic and periprosthetic regimes of [20] differed by 100-fold (5 versus 500 µg/L of each ion). Table 1 shows that whole saliva and biofilm in orthodontic patients differ by a factor of the same order [12], so the orthodontic field should expect the same categorical difference between the programmes engaged in the bulk fluid and at the interface, and should design its experiments as matched regimes rather than as single extracts.

### 5.5. Applicability to Oral Tissues

**Figure 2:**
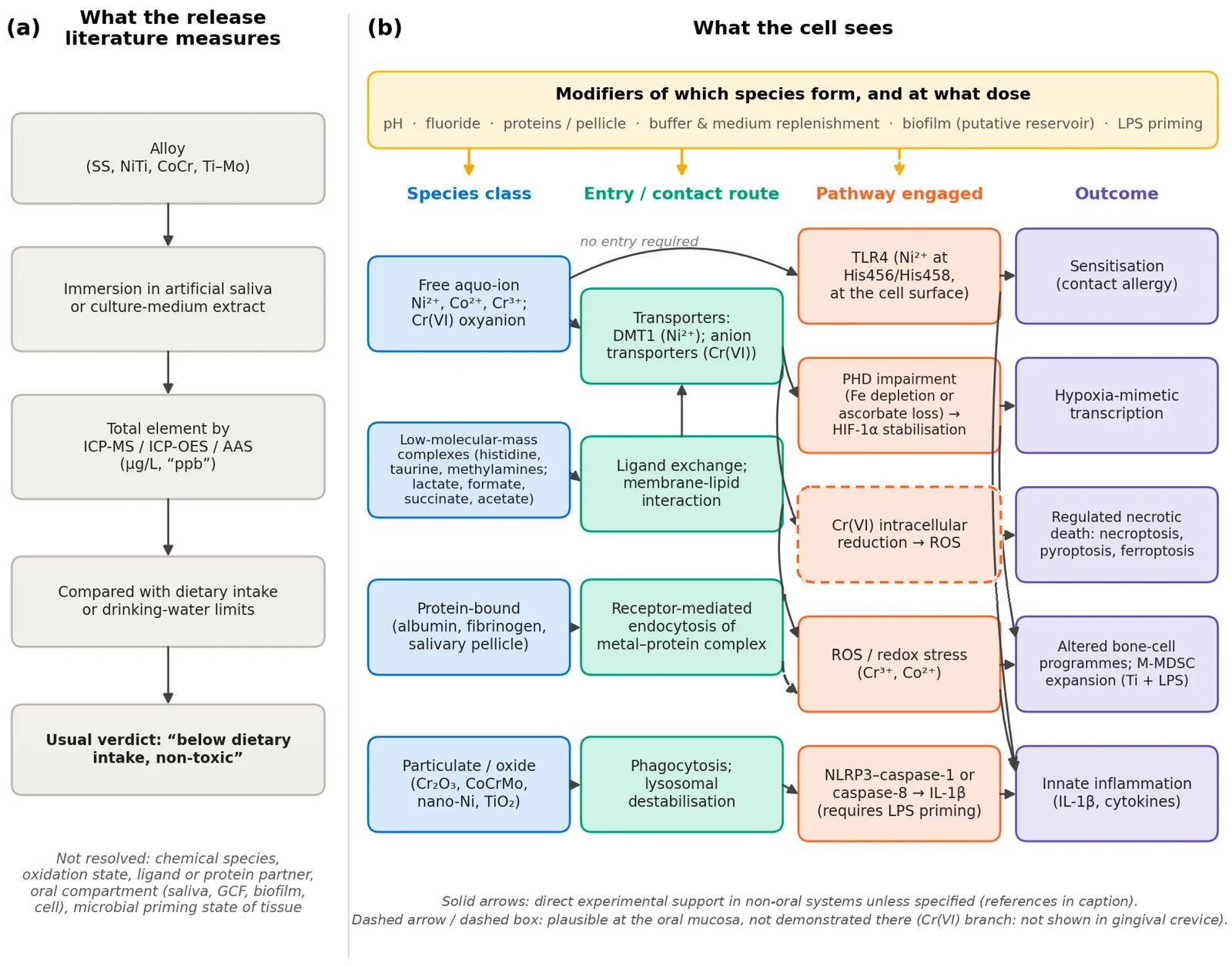
Two views of the same alloy. (**a**) What the release literature measures: the alloy is immersed in artificial saliva or a culture-medium extract, the total element is quantified by ICP-MS, ICP-OES or AAS, and the result is compared with dietary intake or drinking-water limits [2,3,6,11], usually to reach a verdict that release is below dietary intake [3,11], although in [2,6] the same comparison flagged nickel as the element of concern; the chemical species, the oral compartment and the priming state of the tissue are not resolved. (**b**) What the cell sees: a speciation-aware exposure model. Species classes: free aquo-ions and the Cr(VI) oxyanion; low-molecular-mass complexes with histidine, taurine and methylamines at low Ni(II) and with lactate, formate, succinate and acetate carboxylates at higher Ni(II) [18]; protein-bound metal on albumin, fibrinogen and salivary pellicle proteins [26,33,34]; and particulate or oxide forms [43,44]. Entry or contact routes: Ni^2+^ uptake through DMT1 in competition with Fe(II) [37,39]; Cr(VI) uptake through anion transporters with up to 10-fold accumulation [35]; ligand exchange from low-molecular-mass complexes (EDTA-reversible in saliva [18]) and direct metal–membrane-lipid interaction [39]; receptor-mediated endocytosis of specific metal–protein complexes, for which the carrier protein rather than the metal amount sets the delivered dose [40]; and phagocytosis with lysosomal destabilisation of particles [43]. Pathways: Ni^2+^ activates human TLR4 directly at His456/His458 at the cell surface, without cellular entry [41,42]; Ni^2+^-driven iron depletion or ascorbate loss impairs prolyl hydroxylase domain enzymes (PHDs) and stabilises HIF-1α [37,38], a programme also engaged by Co/Cr at periprosthetic concentrations [20] and in gingival cells exposed to Co–Cr extracts [27]; Cr(VI) is reduced intracellularly with ROS generation and Cr(VI)-specific necroptosis, pyroptosis and ferroptosis [46]; Cr^3+^ signals through ROS and Co^2+^ through a caspase-8-dependent, NLRP3-independent route, whereas Cr_2_O_3_ nanoparticles act through cathepsin B and NLRP3–caspase-1 [43]; particulate Ni activates NLRP3 via HDAC6 only in LPS-primed cells [44]. Outcomes: sensitisation and innate inflammation [41,43]; hypoxia-mimetic transcription [20,37]; regulated necrotic death [46]; altered bone-cell programmes [20] and, for Ti ions acting synergistically with LPS, M-MDSC expansion and peri-implant bone loss [45]. The band across the top lists modifiers that change which species form and at what dose: pH [8,21], fluoride [17,19], proteins and pellicle [25,26,34], buffer and medium replenishment [25,26], the biofilm as a putative reservoir [12] (accumulation demonstrated, re-release inferred) and LPS priming [44,45]. Solid arrows denote routes with direct experimental support in non-oral systems unless specified; the dashed arrow (endocytosed metal–protein complexes to intracellular redox signalling) is plausible at the oral mucosa but has not been demonstrated there. The Cr(VI) branch is drawn with a dashed outline: the oxidation of Cr(III) to Cr(VI), and the presence of Cr(VI), have not been demonstrated in the gingival crevice. Most mechanistic evidence originates from skin, lung, kidney, bone-marrow macrophage or orthopaedic systems rather than oral tissue (Table 3, oral evidence level).

None of the studies cited in this section tested oral keratinocytes, gingival fibroblasts or periodontal ligament cells for DMT1, TLR4 or the anion exchangers that carry chromium(VI). Whether these cell types express the transporters and receptors at functional levels, and whether gingival TLR4 shows the same His456/His458 dependence as the receptor studied in skin, is therefore assumed rather than shown, and the routes in Table 3 and Figure 2 are candidates to be tested in oral cells, not established oral mechanisms. Three oral-specific modifiers may further alter both the species and the immune signal: the salivary protein corona and pellicle, which sequester ions and present them as metal–protein adducts rather than aquo-ions [25,26,34]; the biofilm, which accumulates nickel [12] and supplies the microbial priming signal on which inflammasome activation depends [44,45]; and the tolerogenic bias of oral mucosal immunity, which may raise the threshold for sensitisation relative to skin. The last of these has not been examined for alloy-derived metals and is listed as an open question.

Table 3 assembles these findings into a speciation-aware exposure model, mapping each species class to its ligands or physical form, its uptake route, its receptor or pathway and the evidence supporting each link. Figure 2 presents the model schematically. The honesty required of the table is that most rows rest on evidence from skin, lung, kidney, bone or serum rather than oral mucosa. In addition, the oral speciation column rests on one study. The oral evidence level column of Table 3 makes this explicit for every row.

## 6. Reconciling Clinic and Bench

The clinical data are compatible with the model and difficult to reconcile with the total-release hypothesis, subject to the biases set out below. In the systematic review of orthodontic hypersensitivity, concomitant positive allergy tests identified nickel 451 times, cobalt 6, titanium 5 and chromium 4 in a population of 858 subjects aged 9–49, of whom 828 had sex reported (114 males, 714 females) [1]. Stainless steel sheds nickel and chromium together, NiTi sheds nickel and titanium, and titanium alloys in fluoride-rich conditions shed titanium in quantities that dwarf nickel from steel [17]. A ratio of 451:4 between positive nickel and chromium tests is not a ratio of release masses. Much of it is ascertainment bias, since nickel is included in every baseline patch-test series whereas titanium, cobalt and often chromium are not. Much of the remainder is background sensitisation acquired outside the mouth, which varies with sex and jewellery exposure. A receptor-level contribution is plausible given the human-specific His456/His458 site on TLR4 [41], but it cannot be quantitatively apportioned from positivity counts, and no apportionment is attempted here. The included population was six-to-one female; the review reports sex only for the oral manifestations, all eleven with recorded sex being female (M:F = 0:11), and attributes the excess to metal exposure from cosmetics and jewellery, a pattern that is inexplicable by release and points to sensitisation acquired outside the mouth. The prospective lymphocyte-transformation study only partly corroborates this. Half of the start-of-treatment group was nickel-sensitised at some time point, two before bracket placement, both negative three weeks later, and three newly after bracket insertion. The most strongly sensitised individual was an untreated control with minimal salivary nickel. Salivary nickel did not distinguish any group [16]. Sensitisation that does not scale with salivary nickel is compatible with a receptor-limited, species-dependent process. With 30 participants, however, the observation that the most strongly sensitised carried the lowest salivary loads is tentative and is not used here as evidence of mechanism.

The prosthodontic literature shows what happens when exposure is not characterised. A meta-analysis of patch-test-confirmed metal allergy in symptomatic patients with intraoral prostheses and restorations, drawing on 27 articles and 31 case reports, could not pool prevalence: I^2^ was 97.1%, the Q-test *p* < 0.001, and the 95% prediction interval ran from 3% to 91%, across offending metals from nickel, gold and palladium to cobalt, chromium, mercury species, silver, platinum, copper, zinc, tin and indium, with clinical relevance inconsistently reported and study quality medium [48]. Part of that heterogeneity is attributable to differing diagnostic criteria, case selection and patch-test methods rather than to exposure alone; but a field that cannot say whether one symptomatic patient in thirty or nine in ten are affected has, at minimum, not defined its exposure variable. The critical appraisal of systemic nickel allergy syndrome makes the same point from the allergist’s side: the diagnosis rests on small uncontrolled case series without reproducible biomarkers, provocation doses far above dietary intake and neglect of bioavailability. The reported benefit of low-nickel diets is more plausibly a non-specific effect of dietary change such as reduced fermentable carbohydrate [5]. As noted in Section 1, this is argument rather than evidence, but it describes the failure mode that awaits a dental literature that continues to equate total with bioavailable.

At the cellular level, the intracellular measurements confirm the dissociation. The systematic review of oral epithelial cells found metal burden inside the cells accompanied by nuclear alterations that tended to regress [15], and the fluoride trial found that acidulated phosphate fluoride raised intracellular nickel and reduced viability without altering micronucleus frequency [19]. Salivary total, intracellular content, cytotoxicity and genotoxicity moved independently of one another, which is what one expects if each is governed by a different species and route. The in vitro extract literature shows the complementary failure. The band study [21] showed that pH and brand together shaped the verdict. One of two molar bands was cytotoxic relative to control only after acidic ageing and more genotoxic at both pH values, while the other was non-cytotoxic at either, although the direct pH and brand contrasts were not statistically significant. The bracket study applying corrosion eluates from uncoated, nanoparticle-coated and polymer-coated stainless steel, generated in an oral-biomimicry model, to gingival fibroblasts and buccal epithelial cells found no significant difference in cytotoxicity or genotoxicity between the three, although the coated groups showed better corrosion resistance on SEM and reduced cellular differentiation [49]. This null result should not be dismissed as a failed experiment. It shows that an extract assay, presented with the species and concentrations that orthodontic hardware actually generates in a biomimetic model, cannot resolve differences that the surface analysis could. The assay is not wrong. It is answering a question about the wrong species at the wrong concentration, and reporting no difference where the clinic reports 451 positive tests.

The category error is not confined to metals. A quantitative comparison of eight aligner materials exposed human dental-pulp stem cells to eluates at 500, 1000 and 5000 ng per 100 µL for 24–96 h. Thermoformed and polyamide-12 systems kept viability mostly above 70% with preserved morphology. Several directly printed resins approached moderate cytotoxicity at the higher doses and longer exposures, and a subset showed non-linear, hormetic-like dose responses [50]. The exposure was defined as total mass per volume with no identification of the leached species, which is the same error in a different chemistry, and the non-monotonic responses are inconsistent, within a single assay, with the linear assumption that more release means more harm. The point is made and will not be extended to photoinitiators or particulates here.

## 7. Hazard, Risk and the Regulatory Frame

The regulatory frame already contains the distinction that the release literature collapses. Regulation (EU) 2017/745, Annex I, Chapter II, Section 10.4.1, permits substances classified as carcinogenic, mutagenic or toxic to reproduction (CMR) of category 1A or 1B, or with endocrine-disrupting properties, above 0.1% w/w in devices that are invasive and come into direct contact with the human body only where justified under Section 10.4.2 of the same Annex [51]. Among alloy constituents, nickel compounds (Carc. 1A) and cobalt metal (Carc. 1B) carry such classifications, whereas nickel metal (Carc. 2) does not. This is a compositional, hazard-based criterion. It says nothing about release, and it is satisfied or breached by an alloy’s certificate of analysis. Exposure-based risk assessment lives in a different instrument, ISO 10993-17:2023 [52], whose tolerable-intake machinery has recently been tested against patient populations. Chemical-specific tolerable-intake values for 1213 constituents derived under the standard’s 2023 default assumptions were compared with body-weight-adjusted toxicological screening limits and thresholds of toxicological concern. These were protective for adults, but for 10-kg children and 3.5-kg infants the chemical-specific values were up to ten-fold lower than the adjusted screening values for up to 5.9% of constituents at exposures under one year, and the screening limit formally excludes infants under six months [53]. The authors nevertheless judge that once the conservatism of the default tolerable-intake derivation is taken into account, the screening limits are likely to be adequately protective above six months of age. Fixed-appliance treatment typically begins in adolescence [1], a body-weight range for which the adult-calibrated screening has simply not been evaluated. The shortfall shown for 10-kg and 3.5-kg body weights does not transfer directly to orthodontic patients, but it shows that default scaling is not automatically protective below adult body weight.

Between hazard classification and tolerable intake sit two further tiers that are routinely conflated. Total release measured in an immersion study is a property of a material–medium pair, and as the inter-laboratory study showed it can differ by up to 240% between laboratories using the same standardised extraction conditions [29]. The bioaccessible fraction is narrower than the total and depends on the medium, as the tattoo-ink study demonstrated with nickel solubility differing significantly among deionised water, saline and artificial sweat (up to 63% in saline) in a regulatory setting that limits soluble fractions without prescribing the medium [30]. The dental-device space has the identical gap. The interfacial species is narrower still: it is the subset of the bioaccessible fraction that exists in a form with a route into the target cell. The tiers cannot be converted into one another without assumptions that are presently unavailable or unverified; what can be done, and is done in Supplementary Section S1, is a sequential calculation under stated assumptions, which quantifies the gap rather than closing it. A tolerable intake is derived for an element or a defined compound entering the systemic circulation, and an extract concentration cannot be mapped onto it without assuming a species, an absorption route and a compartment volume, none of which the extract specifies. A bioaccessible fraction measured in one medium cannot be carried to another, since the tattoo-ink data show it changing with the medium; and no interfacial species has been measured at all. Under the assumptions of Section S1, measured crevicular nickel (19.8–101.8 µg/L [13,14]) lies 4- to 20-fold above the systemic regime and 5- to 25-fold below the periprosthetic regime at which HIF-1α signalling was engaged in bone cells [20], a comparison that already crosses elements (nickel against cobalt and chromium) and tissues. With an uptake fraction of 10%, the effective intracellular supply falls to the systemic regime. The biofilm scraping data of [12] (21.58 µg/L in a 0.5 mL suspension of a 3 mm^2^ scraping, i.e., ≈11 ng per scraping) imply an in situ concentration of ≈18–72 mg/L if the biofilm is 200–50 µm thick, which lies within or above the ^1^H NMR titration window [18]. This is the one compartment in which the speciation data may already be at the right concentration, but the estimate changes four-fold with the assumed thickness and says nothing about what fraction of biofilm nickel is in a fluid phase. The NLRP3 data of [44] are expressed as a dose per animal and could not be converted to a concentration. Each of these numbers depends on an assumption that no study has measured, which is the operational meaning of the gap.

These are five different evidentiary tiers, and each licenses a different statement. Hazard classification licenses “this alloy contains a substance of concern above a threshold”. Total release licenses “under these conditions this much left the surface”. Tolerable intake licenses “a systemic dose of this size would be acceptable in an adult”. Bioaccessible fraction licenses “this proportion could enter solution in this medium”. Only the interfacial species licenses a statement about biological effect at the mucosa, and only one orthodontic release study has approached it, by separating chromium(VI) from chromium(III) in a bracket extract [36]. None has measured a species at the mucosa. The dietary-comparison lineage that runs from Brune’s “standard restored man” [2] through Huang’s “below daily dietary intake” [3] to the 2026 conclusion that post-debonding levels “can be considered non-toxic” [11] takes a measurement from the second tier and phrases a conclusion from the third, while the effect it purports to exclude belongs to the fifth. The lineage was reasonable in 1986, when the alternative was no comparison at all, and the comparison retains its place as a screen for systemic exposure. As a way of excluding local effects forty years later, it should be retired. The author’s own contribution to it [6], which used the same comparison even though the author identified nickel as a risk, is included in that verdict.

## 8. A Speciation-Aware Research Agenda

The remedy is not more immersion studies but a change in what they report. Table 4 lists a minimum reporting set for release studies and a set of prioritised experiments. The reporting set is intended to make existing designs interpretable and the experiments to reach the fifth evidentiary tier.

The first priority is speciation modelling. The thermodynamic approach that offered an explanation for aluminium patch-test variability, and for the pH shifts caused by aluminium and chromium salts, by computing bioavailable fraction and form as functions of salt, concentration, sweat composition and pH [32] can be applied to every artificial-saliva recipe compiled in the corrosion guide [24] and to real saliva using the ligand inventory and stability-constant hierarchy that the NMR titration established [18]. The output would be a species distribution rather than a total, and it would reveal immediately which recipes deliver aquo-ion, which deliver carboxylate complexes and which precipitate the metal as phosphate. The second is a protein-containing, renewed medium as the default, given that proteins suppress nickel and chromium release from NiTi and steel [25] but enhance cobalt, chromium and molybdenum release from CoCrMo, suppress the wear-track acidification seen without proteins and constrain the buffer choice [26]; a protein-free static bath measures a reaction that does not occur in the mouth.

The third is sampling the compartments that carry the dose: gingival crevicular fluid, where the two prospective studies that report it in secure units place nickel between the pooled whole-saliva increments and ten times above them [10,13,14], and where the unit error corrected in [13] shows how easily a compartment can be misread by three orders of magnitude, and biofilm, which holds nickel at a thousandfold the salivary level after 9–12 months [12], followed by speciation of both, including a direct test of whether chromium(III) is oxidised to chromium(VI) in crevicular fluid. The fourth is intracellular metal in buccal cells as a routine endpoint, following the randomised fluoride trial that measured it [19]. The fifth is matched concentration regimes in cell models. The transcriptomic comparison of systemic and periprosthetic cobalt and chromium [20] should be replicated with salivary and crevicular concentrations on oral keratinocytes, gingival fibroblasts and periodontal ligament cells. The sixth is primed versus unprimed cell models, because particulate nickel requires LPS priming to engage NLRP3 [44] and titanium ions act synergistically with LPS on myeloid suppressor cells [45]. An assay on resting cells in serum-free medium models a mucosa without a microbiome. The seventh is a paediatric tolerable-intake derivation, since the default screening has not been evaluated for adolescent body weights and is least conservative for a small fraction of constituents in children [53], and the orthodontic population is precisely that part of the public.

One direction is offered as an untested hypothesis. Orthodontic tooth movement is an inflammatory, hypoxia-sensitive remodelling of the periodontal ligament and alveolar bone. Cobalt and chromium at systemic-circulating concentrations inhibit osteoclast differentiation, and at periprosthetic concentrations engage HIF-1α in osteoblasts [20]. Nickel produces a pseudo-hypoxic, HIF-1α-stabilising state through impairment of prolyl hydroxylases [37,38]. Whether the ions an appliance sheds into the crevice modify the rate or quality of tooth movement has not been examined, and the present model predicts that any such effect would depend on species and compartment rather than on total release. It is a hypothesis and no more.

### Limitations of This Review

This review has limitations of its own. Sources were selected by a single author for their bearing on the argument rather than through a systematic search protocol, with a preference for primary studies published in 2021–2026 and for the original report of each mechanism. No claim of completeness is made and no quantitative synthesis was attempted. The absence of a formal search protocol, screening log and record counts (see “Scope, Source Selection and Limits of the Evidence Base” in Section 1) means that the evidentiary gaps identified in Table 3 and Table 4 are the author’s judgement and could reflect selective citation. Most of the mechanistic evidence in Section 5 and Table 3 is transferred from skin, lung, kidney, bone-marrow macrophage and orthopaedic systems, and its applicability to oral mucosa is inferred rather than demonstrated. The crevicular data rest on two studies, one of which had its unit corrected after publication, and no study has measured whole-saliva and crevicular metal in the same patients. The oxidation of chromium(III) to chromium(VI) in the gingival crevice, on which the chromium branch of the model depends, has not been tested. Finally, the speciation of alloy-derived metals in saliva rests on two titration studies whose lowest concentrations lie well above the whole-saliva values reported in patients, and on one chromium(VI) separation in synthetic saliva. Section 4 and Section 5 and Table 3 are accordingly a hypothesis-generating framework. The nickel(II) speciation data were obtained at concentrations 41- to 360-fold above those measured in patients and are not extrapolated to the patient range.

## 9. Conclusions

The orthodontic and prosthodontic literature on metal release has measured the right elements in the wrong form and compared them with the wrong reference. Totals of nickel, chromium, cobalt, molybdenum and titanium in artificial or whole saliva are properties of a material–medium pair; they have not been shown to be reproducible between laboratories. The only relevant inter-laboratory data come from standardised polymer extraction, where results could differ by up to 240% [29]. The results vary with the medium by orders of magnitude, and their relationship to concentrations at the gingival crevice, in the biofilm or inside the epithelial cell, is unknown, no study having measured them together. Comparing them with dietary intake is a legitimate screen for systemic exposure, but used to exclude local effects it conflates a local interfacial exposure with a systemic dose and uses a measurement from one evidentiary tier to phrase a conclusion from another.

Where it has been tested, biological effect has proved to be a function of species rather than of total element. The same element as aquo-ion, low-molecular-mass complex, protein adduct, oxyanion, nanoparticle or micron particle enters cells by different routes, through DMT1, membrane interaction, receptor-mediated endocytosis, anion transport or phagocytosis, and engages different receptors and pathways, from TLR4 coordination at His456/His458 to NLRP3- or caspase-8-dependent inflammasome routes, prolyl-hydroxylase inhibition and HIF-1α. The chromium branch of the model stands on weaker ground and is not placed alongside these: it depends on hexavalent chromium reaching the gingival crevice, which has not been demonstrated, and on a single unreplicated separation in synthetic saliva [36]. Several of these effects are conditional on microbial priming and on concentration regime, so that the same dose is inert in one context and active in another.

The evidence for these statements is strong in skin, lung, kidney, bone and serum and thin in the mouth, where salivary speciation rests on two NMR titrations. That asymmetry is not a reason to retain the dietary comparison. It is the reason to replace it. A speciation-aware exposure model, a minimum reporting set and seven prioritised experiments are proposed. Until release studies report species, medium, compartment and intracellular dose, the statement that a released quantity is below dietary intake should be recognised for what it is: true, and beside the point.

## Figures and Tables

**Table 2:** Protocol variables that change the species released, not only the amount. Evidence from orthodontic and prosthodontic alloys and, where marked, from adjacent regulated fields. Sources: [8,9,17,19,21,23,24,25,26,27,29,30].

Variable	Direction of Effect	Magnitude/Evidence	Consequence for Speciation or Exposure	Ref.
pH of medium	Lower pH increases Ni and Ti release from NiTi (28 d, pH 4.8–6.6)	Ni/Ti release inversely proportional to pH; Ni–pH relation in uncoated wires only; roughness unaffected, hardness/modulus marginally affected	Acidity governs release without surface change; authors still hedge that hypersensitivity cannot be excluded	[8]
pH of ageing medium (band extracts)	pH 4.5 flips the cytotoxicity verdict for one of two commercial molar-band brands	Brand AO cytotoxic only at pH 4.5 (*p* = 0.04); both brands DNA damage at pH 4.5	Extract at one pH answers a different question from the same device at another pH	[21]
Fluoride concentration	Threshold, not linear, effect at 380 ppm F for Ti-containing wires	SS ~500 ppb Ni, ~1000 ppb Cr; NiTi >220,000 ppb Ni, >180,000 ppb Ti; pitting, martensite plates, Ni-rich precipitates	Two to three orders of magnitude; abrupt regime change at 380 ppm F—a change of chemistry, not a scaling	[17]
Fluoride product plus prior intraoral use	Used appliances and 1.23% APF increase release, roughness and cytotoxicity	Used > new for ions, roughness and fibroblast cytotoxicity; APF greatest viability loss in the TMA subgroup	New-appliance extracts underestimate clinical exposure; DMEM extract differs from saliva	[23]
Fluoride product in patients (intracellular endpoint)	APF raises intracellular buccal-cell Ni; neutral gel and varnish do not	Ni ↑ only in APF arm (*p* < 0.05); Cr unchanged; viability ↓; micronuclei unchanged	A speciation modifier changes cellular uptake and viability without changing genotoxicity	[19]
Proteins in medium (orthodontic appliance)	Protein-containing YPD suppresses Ni and Cr, promotes Fe, Cu and Al leaching	Ni/Cr species in top surface layer on NiTi in artificial saliva but not in YPD (XPS)	Protein-free artificial saliva mis-estimates both the elements and the surface species released	[25]
Proteins in medium (CoCrMo)	10 g/L BSA + 2.5 g/L fibrinogen strongly enhance Co, Cr and Mo release, yet lower tribocorrosion	Protein-free reference showed Mo oxidation and local acidification in wear track; abolished by proteins	Proteins raise the amount and change the surface-oxide and wear-track chemistry simultaneously	[26]
Buffer choice	MES buffer was required to avoid metal–phosphate and metal–protein aggregate precipitation	Buffer had to be MES, not phosphate, to keep metal in solution	The buffer decides whether released metal is measured as dissolved or lost as precipitate	[26]
Static vs. renewed medium	Replenishment regime alters surface chemistry and leaching	Two regimes compared by ICP-MS, XPS, SEM/EDX, AFM	In static artificial saliva, a protective layer formed and eluted ions fell after day 14, whereas replenishment gave continuous leaching; the regime changes the result	[25]
Component and welding; as-received pitting	SS bands release an order of magnitude more than other parts	Pitting already present on as-received brackets/bands; KCl precipitation; no correlation with roughness	Whole-appliance totals hide component-resolved sources; “no clear conclusions” due to interacting factors	[9]
Manufacturing route/recycled powder	Recycled Co–Cr powder (SLM) increases ion release and cytotoxicity	Concentration-dependent apoptosis, ROS, HIF-1α, TNF-α/IL-6/IL-8/VEGF in gingival cells (DMEM extracts)	Nominally identical alloy composition yields different release and response	[27]
Recipe heterogeneity of simulated fluids	Formulations differ in pH, halide, Ca/P, proteins, gas control and flow	No standardised fluoride–pH matrix; limited guidance for microbiome-integrated assays	Quantitative links between simulated-fluid testing and clinical outcome “insufficiently established”	[24]
Inter-laboratory reproducibility of standardised extraction ^a^	Same ISO 10993-18 conditions, eight laboratories	Reproducibility RSD central 90% range 0.30–0.85; results could differ by up to 240% (95% confidence, 95% of systems)	Even under identical standardised conditions, results could differ between laboratories by up to 240%	[29]
Extraction medium vs. bioaccessible fraction ^a^	Bioaccessibility depends significantly on medium (*p* < 0.05)	Ni 63% soluble in saline, 59% in artificial sweat (black inks); Hg up to 72%	Limits set on soluble fractions without a prescribed medium—the same gap as for saliva	[30]

^a^ Evidence from adjacent regulated fields (medical device polymer extraction [29]; tattoo inks [30]), not from dental alloys; transferred here as methodological analogues. ↑, increase; ↓, decrease. Abbreviations are defined in the list at the end of the article.

**Table 3:** A speciation-aware exposure model, presented as a hypothesis-generating framework: species class, dominant form, uptake route, signalling, evidence base and oral evidence level. The compartment of origin of each mechanistic study is stated explicitly; most mechanisms are transferred from skin, lung, kidney, bone-marrow macrophage or orthopaedic systems, and only [18,31,34] derive from human saliva. Sources: [18,20,26,27,31,32,33,34,35,36,37,39,40,41,42,43,44,45,46].

Species Class	Dominant Form/Ligands in the Oral Environment	Cellular Entry Route	Receptor/Pathway Engaged	Key Evidence (Compartment of Origin)	Oral Evidence Level (Direct/Indirect/Absent)	Ref.
Free aquo-ion Ni^2+^	Labile hydrated ion; the fraction not captured by salivary ligands (proportion in saliva not reported)	DMT1, competing with Fe(II); direct membrane-lipid interaction	Human TLR4 via His456/His458 (non-LPS agonist); Fe displacement → aconitase/IRP1, pseudo-hypoxic HIF-1α	TLR4: transgenic mouse skin, human receptor; 8 h stromal response in human skin; DMT1: DMT1-overexpressing HEK (embryonic kidney) vs. A549 lung cells (~40% Fe loss); membrane route: review	Absent (TLR4: human receptor in skin and transgenic mouse skin; DMT1: HEK/A549 cells)	[37,39,41,42]
Low-molecular-mass Ni(II) complexes	Histidine and methylamines (N-donors) at 71 µM added Ni; lactate, formate, succinate, acetate (O-donors) at 140–670 µM; EDTA-reversible	not reported (transferable complexes; route not measured)	not reported; speciation modelling links bioavailable form to pH, ligand and concentration	^1^H NMR titrations of human salivary supernatants (Ni(II), *n* = 12; Co(II)); speciation modelling of Al/Cr salts on skin (patch tests)	Direct for species identity (ex vivo human saliva, added Ni/Co at ≥71 µM); absent for uptake route	[18,31,32]
Protein-bound metal (albumin, pellicle proteins)	396 salivary proteins adsorb to Ti (369) and ceramic (298); BSA/fibrinogen enhance Co, Cr, Mo release; MES rather than phosphate buffer needed to avoid metal–phosphate/metal–protein precipitation; Co(II)/Cr(III) aggregate albumin	Receptor-mediated endocytosis of the specific metal–protein complex (megalin:cubilin archetype)	Carrier protein, not metal quantity, sets delivered dose and toxicity; immune-process hub proteins in the adsorbed pellicle	Pellicle proteomics on Ti (pooled human saliva); CoCrMo corrosion with proteins (in vitro); BSA aggregation (in vitro); Cd–protein endocytosis (rat renal proximal tubule)	Indirect (pellicle proteomics on Ti from pooled human saliva); absent for endocytic route	[26,33,34,40]
Cr(III) vs. Cr(VI)	Alloys shed Cr(III); Cr(VI) forms on H_2_O_2_ oxidation at pH 7.4 (mineral matrix)	Cr(VI): anion transporters (up to 10-fold accumulation; inhibitors reduce uptake up to 45-fold); Cr(III): poorly membrane-permeant; uptake route not established	Cr(VI): RIPK1/RIPK3 necroptosis, NLRP3/GSDMD pyroptosis, ferroptosis; Cr^3+^: ROS → NLRP3/caspase-1 IL-1β	Cr(VI) uptake: epithelial/mesothelial/carcinoma lines (asbestos model); death pathways: review; Cr^3+^: LPS-primed mouse bone-marrow macrophages	Indirect (Cr(VI) separated from a bracket extract in synthetic saliva [36], unreplicated); absent in GCF/biofilm and for all pathways	[35,36,43,46]
Co^2+^	Divalent ion in DMEM extracts or defined salts; ratio to Cr shapes response	Transporters and membrane-lipid interaction (Co/Ni/Mn shared homeostasis)	Caspase-8-dependent, NLRP3-independent IL-1β; HIF-1α stabilisation, TNF-α/IL-6/IL-8/VEGF; regime-dependent osteoblast/osteoclast programmes	LPS-primed mouse bone-marrow macrophages; human gingival fibroblasts/keratinocytes (Co–Cr DMEM extract); primary human bone cells (orthopaedic concentrations)	Indirect (human gingival fibroblasts/keratinocytes exposed to Co–Cr DMEM extract); absent for caspase-8/NLRP3 route	[20,27,39,43]
Particulate/oxide	Cr_2_O_3_ nanoparticles (60 nm); CoCrMo particles (3.4 µm); Nano-Ni; CoCr nanoparticles	Phagocytosis/lysosomal uptake (cathepsin B release for Cr_2_O_3_)	Cr_2_O_3_: NLRP3/caspase-1 via lysosomal destabilisation; CoCrMo particles: caspase-8; Nano-Ni: HDAC6 → NLRP3 only after LPS priming	LPS-primed mouse bone-marrow macrophages; mouse lung and MH-S alveolar macrophages (50 µg intratracheal); bone cells (orthopaedic)	Absent	[20,43,44]
Ti ions vs. Ti particles	Ti ions from fluoride-attacked or corroding Ti alloys (Ti–F complexes destabilise TiO_2_); particle branch: see above	not reported for Ti ions	Ti ions + LPS synergistically expand M-MDSCs; c-Fms (CD115) upregulation; peri-implant bone loss	Mouse bone-marrow precursors and ligature-induced peri-implantitis (oral tissue)	Indirect (mouse ligature peri-implantitis model; no human oral data)	[45]

Compartments of origin are stated as in the sources; no oral-mucosal study directly demonstrates the TLR4, DMT1, megalin:cubilin or anion-transporter routes for orthodontic alloys. “Not reported” indicates that the source contains no information on that cell. Oral evidence level: direct = the stated link demonstrated in human oral tissue or saliva; indirect = oral cells, oral-derived material or an animal oral model under non-physiological conditions; absent = no oral data for the stated link. →, leads to (mechanistic sequence). Abbreviations are defined in the list at the end of the article.

**Table 4:** Minimum reporting set and prioritised experiments for speciation-aware release studies. Each item is derived from a documented failure mode in the cited evidence. Sources: [1,2,9,12,13,14,15,16,18,19,20,24,25,26,29,30,31,32,36,44,45,53].

Domain	Typical Design of the Cited Studies (Author’s Characterisation)	Minimum Reporting Item Proposed	Rationale/Evidence	Ref.
Medium composition	Protein-free artificial saliva of unstated or variable recipe; buffer rarely justified [3,6,7,8,9,17,21,23]	Full recipe in g/L; protein content; buffer identity; fluoride (ppm); pH at start and end	Recipes heterogeneous with no fluoride–pH matrix; proteins suppress Ni/Cr but promote Fe/Cu/Al; phosphate buffer precipitates metal	[24,25,26]
Replenishment regime	Static immersion for days to weeks [3,6,7,8,9,17]	State static vs. renewed; renewal interval and volume; report both regimes where feasible	Static vs. renewed medium altered surface chemistry and leaching of NiTi/SS appliance	[25]
Surface area and component resolution	Whole appliance or unspecified assembly; totals per litre [3,6,7,8,17]	Release per cm^2^ and per component; welding/joint status; as-received surface (SEM) documented	Bands released an order of magnitude more (welding); pitting on as-received parts; 1986 “standard restored man” normalisation	[2,9]
Speciation	Total element by ICP/AAS only [3,6,7,8,9,10,11,12,13,14,17]	Modelled speciation (thermodynamic speciation software, name and version stated) of the medium and, where possible, measured species (e.g., ^1^H NMR)	Speciation modelling offers an explanation for patch-test variability; salivary Ni and Co partition among N-donor and carboxylate ligands by concentration; ~40% of Cr released from brackets in synthetic saliva recovered as Cr(VI)	[18,31,32,36]
Bioaccessible fraction	Not distinguished from total content [3,6,7,8,9,17]	Report soluble/bioaccessible fraction in a stated medium alongside total	Ni bioaccessibility up to 63% (saline) and 59% (artificial sweat) in black inks; significant medium effect across the three media (*p* < 0.05)	[30]
Interfacial compartments	Whole saliva only [10,11,16,19]	Sample GCF and biofilm alongside saliva; report per-compartment values and ratios	GCF Ni 3.894 → 19.810 µg/L at 6 months (unit corrected by erratum) and ~102 µg/L at 30 d under fluoride; biofilm Ni ~1000× other compartments at 9–12 months	[12,13,14]
Intracellular dose	Salivary concentration used as surrogate for exposure [10,11,13,14,16]	Metal content of buccal epithelial cells (ICP-MS) with cytological endpoints	Intracellular Ni ↑ only in APF arm without micronucleus change; epithelial metal burden and nuclear alterations reported across a 19-study systematic review	[15,19]
Concentration regimes in cell studies	Single or arbitrary concentration; extract of undefined ion content [21,23,27,49]	Test at least two regimes matched to measured compartments (saliva µg/L vs. GCF/biofilm) and state species tested	Systemic vs. periprosthetic Co/Cr regimes produced categorically different transcriptional programmes (HIF-1α at local levels)	[20]
Primed vs. unprimed cell context	Unprimed monocultures without microbial signal [21,23,27,49]	Include LPS-primed (or biofilm-conditioned) arm; report priming status	Nano-Ni activates NLRP3 only in LPS-primed cells; Ti ions and LPS synergistically expand M-MDSCs	[44,45]
Reference dose framework	Comparison with dietary intake or drinking-water limits [2,3,6,8,11]	Chemical-specific tolerable intake with paediatric body weight (10 kg child, 3.5 kg infant) stated	Chemical-specific TI values up to 10-fold below body-weight-adjusted TSL/TTC for up to 5.9% of constituents at exposures <1 year; authors judge the difference likely conservative above six months; adolescent body weights not evaluated	[53]
Reproducibility statement	Single laboratory, replicates unstated [3,6,7,8,9,17,25]	Detection limit, recovery, repeatability/reproducibility RSD; inter-laboratory comparison where standard extracts are used	Same ISO 10993-18 conditions across eight laboratories implied inter-laboratory differences of up to 240%	[29]
Clinical outcome linkage	Release reported without patient endpoint [3,6,7,8,9,17,25]	Pair release data with patch test/LTT or clinical reaction data; report sex distribution	451 Ni:6 Co:5 Ti:4 Cr positive tests, 6:1 female sample; sensitisation not proportional to salivary nickel, group means indistinguishable	[1,16]

“Typical design of the cited studies” characterises the designs of the specific studies cited in each entry (and, for release studies, those of Table 1 and Table 2). It is the present review’s characterisation of those studies, not a quantitative survey of the field. →, change from baseline to the time point stated; ↑, increase. Abbreviations are defined in the list at the end of the article.

## Data Availability

No new experimental data were created. The harmonised values in Table 1 were computed from published data as described in the table footnotes.

## Supplementary Materials

The following supporting information can be downloaded at: https://www.mdpi.com/article/10.3390/ijms27209318/s1, References [2,3,6,8,10,11,12,13,14,17,18,20,41,44] are cited in the Supplementary Materials.

## Abbreviations

The following abbreviations are used in this manuscript:

AAS	atomic absorption spectrometry
AFM	atomic force microscopy
APF	acidulated phosphate fluoride
BSA	bovine serum albumin
CCL21	C-C motif chemokine ligand 21
CCR7	C-C chemokine receptor type 7
CI	confidence interval
CMR	carcinogenic, mutagenic or toxic to reproduction
CoCrMo	cobalt–chromium–molybdenum alloy
DMEM	Dulbecco’s modified Eagle’s medium
DMT1	divalent metal transporter 1
EDTA	ethylenediaminetetraacetic acid
EDX	energy-dispersive X-ray spectroscopy
FDA	United States Food and Drug Administration
GCF	gingival crevicular fluid
GF-AAS	graphite-furnace atomic absorption spectrometry
GSDMD	gasdermin D
HDAC6	histone deacetylase 6
HEK	human embryonic kidney (cells)
HIF-1α	hypoxia-inducible factor 1-alpha
ICP	inductively coupled plasma
ICP-MS	inductively coupled plasma mass spectrometry
ICP-OES	inductively coupled plasma optical emission spectrometry
IL	interleukin
IRP1	iron-regulatory protein 1
ISO	International Organization for Standardization
KLF2	Krüppel-like factor 2
LPS	lipopolysaccharide
LTT	lymphocyte transformation test
MD	mean difference
MES	2-(N-morpholino)ethanesulfonic acid
M-MDSC	monocytic myeloid-derived suppressor cell
MTT	3-(4,5-dimethylthiazol-2-yl)-2,5-diphenyltetrazolium bromide assay
NiTi	nickel–titanium alloy
NLRP3	NOD-like receptor family pyrin domain-containing 3
NMR	nuclear magnetic resonance
PRISMA	Preferred Reporting Items for Systematic Reviews and Meta-Analyses
RCT	randomised clinical trial
RIPK	receptor-interacting protein kinase
RM-ANOVA	repeated-measures analysis of variance
ROS	reactive oxygen species
RSD	relative standard deviation
SEM	scanning electron microscopy
SLM	selective laser melting
SS	stainless steel
TI	tolerable intake
TLR4	Toll-like receptor 4
TMA	titanium–molybdenum alloy
TNF-α	tumour necrosis factor alpha
TSL	toxicological screening limit
TTC	threshold of toxicological concern
VEGF	vascular endothelial growth factor
XPS	X-ray photoelectron spectroscopy
YPD	yeast extract–peptone–dextrose

## References

[1] Amato A., Martina S., De Benedetto G., Michelotti A., Amato M., Di Spirito F. (2025). Hypersensitivity in Orthodontics: A Systematic Review of Oral and Extra-Oral Reactions. J. Clin. Med..

[2] Brune D. (1986). Metal release from dental biomaterials. Biomaterials.

[3] Huang H.-H., Chiu Y.-H., Lee T.-H., Wu S.-C., Yang H.-W., Su K.-H., Hsu C.-C. (2003). Ion release from NiTi orthodontic wires in artificial saliva with various acidities. Biomaterials.

[4] Stoyanova-Ivanova A., Georgiev V., Martins J.N.R. (2025). Nickel Ion Release in Nickel-Containing Orthodontics Archwires: A Narrative Review of In Vitro and In Vivo Studies. Dent. J..

[5] Giovannetti A. (2026). Systemic Nickel Allergy Syndrome: A Critical Appraisal of an Unvalidated Diagnostic Entity. Clin. Transl. Allergy.

[6] Mikulewicz M., Chojnacka K., Woźniak B., Downarowicz P. (2011). Release of metal ions from orthodontic appliances: An in vitro study. Biol. Trace Elem. Res..

[7] Mikulewicz M., Suski P., Tokarczuk O., Warzyńska-Maciejewska M., Pohl P., Tokarczuk B. (2024). Metal Ion Release from Orthodontic Archwires: A Comparative Study of Biocompatibility and Corrosion Resistance. Molecules.

[8] Jusufi Osmani Z., Poljšak B., Zelenika S., Kamenar E., Marković K., Perčić M., Katić V. (2022). Ion Release and Surface Changes of Nickel-Titanium Archwires Induced by Changes in the pH Value of the Saliva—Significance for Human Health Risk Assessment. Materials.

[9] Petković Didović M., Jelovica Badovinac I., Fiket Ž., Žigon J., Rinčić Mlinarić M., Čanadi Jurešić G. (2023). Cytotoxicity of Metal Ions Released from NiTi and Stainless Steel Orthodontic Appliances, Part 1: Surface Morphology and Ion Release Variations. Materials.

[10] Imani M.M., Mozaffari H.R., Ramezani M., Sadeghi M. (2019). Effect of Fixed Orthodontic Treatment on Salivary Nickel and Chromium Levels: A Systematic Review and Meta-Analysis of Observational Studies. Dent. J..

[11] Valdez-Vargas B.J., Lara-Carrillo E., Rubio-Rosas E., Hegazy-Hassan W., Toral-Rizo V.H., Salmerón-Valdés E.N., Osorio-Bernal I.M., Sicco E., Bologna-Molina R., Guzmán-Celaya G.E. (2026). Salivary Cobalt, Nickel, Chromium and Titanium After Fixed Orthodontic Appliance Removal: A 3-Month Pilot Longitudinal Study. Dent. J..

[12] Galviz-García L., Romero-Romero S., Herrera-Herrera A., Mercado-Camargo J., Díaz-Caballero A., Plazas-Román J., Ardila C.M. (2025). Nickel Bioaccumulation in Oral Biofilm, Gingival Tissue, and Saliva During Fixed Orthodontic Treatment: A 12-Month Prospective Cohort Study on Predictors and Salivary pH Correlation. J. Clin. Exp. Dent..

[13] Amini F., Shariati M., Sobouti F., Rakhshan V. (2016). Effects of fixed orthodontic treatment on nickel and chromium levels in gingival crevicular fluid as a novel systemic biomarker of trace elements: A longitudinal study. Am. J. Orthod. Dentofac. Orthop..

[14] Chitra P., Prashantha G.S., Rao A. (2019). Long-term evaluation of metal ion release in orthodontic patients using fluoridated oral hygiene agents: An in vivo study. J. World Fed. Orthod..

[15] Modugno F.P., Kuhn E., Bianchi C.L., Runza L., Pellegrini M., Pulicari F., Spadari F. (2025). Biological Consequences and Assessment Methods Analysis of Fixed Orthodontic Appliances on Oral Epithelial Cells: A Systematic Review. J. Oral Pathol. Med..

[16] Paschaei N., Müller W.-D., Schmidt F., Hüsker K., von Baehr V., Pandis N., Jost-Brinkmann P.-G., Bartzela T. (2024). Unveiling the Role of Metal Ion Concentration versus Immune Sensitization in Orthodontic Patients—A Long-Term Prospective Evaluation. J. Clin. Med..

[17] Pastor F., Rodriguez J.C., Barrera J.M., García-Menocal J.A.D., Brizuela A., Puigdollers A., Espinar E., Gil J. (2023). Effect of Fluoride Content of Mouthwashes on the Metallic Ion Release in Different Orthodontics Archwires. Int. J. Environ. Res. Public Health.

[18] Hunwin K., Page G., Edgar M., Bhogadia M., Grootveld M. (2025). Speciation of Potentially Carcinogenic Trace Nickel(II) Ion Levels in Human Saliva: A Sequential Metabolomics-Facilitated High-Field ^1^H NMR Investigation. Metabolites.

[19] Apiwantanakul N., Chantarawaratit P.-O. (2021). Cytotoxicity, genotoxicity, and cellular metal accumulation caused by professionally applied fluoride products in patients with fixed orthodontic appliances: A randomized clinical trial. J. World Fed. Orthod..

[20] Shah K.M., Dunning M.J., Gartland A., Wilkinson J.M. (2021). Distinct Concentration-Dependent Molecular Pathways Regulate Bone Cell Responses to Cobalt and Chromium Exposure from Joint Replacement Prostheses. Int. J. Mol. Sci..

[21] Karandish M., Khademi B., Ziaei G., Erfani N., Moezi L., Faghih Z. (2025). Cytotoxicity and genotoxicity of orthodontic bands after aging: An in-vitro study. BMC Oral Health.

[22] Nakagawa M., Matsuya S., Shiraishi T., Ohta M. (1999). Effect of fluoride concentration and pH on corrosion behavior of titanium for dental use. J. Dent. Res..

[23] Chantarawaratit P.-O., Yanisarapan T. (2021). Exposure to the oral environment enhances the corrosion of metal orthodontic appliances caused by fluoride-containing products: Cytotoxicity, metal ion release, and surface roughness. Am. J. Orthod. Dentofac. Orthop..

[24] Bordbar-Khiabani A. (2026). Simulated Body Fluids for Dental Implant Corrosion: A Practical Guide. Dent. J..

[25] Petković Didović M., Kara K., Jelovica Badovinac I., Peter R., Fiket Ž., Suman I., Kowalkowski T., Čanadi Jurešić G. (2025). Metal release profiles of NiTi and stainless steel orthodontic appliances: The influence of proteinaceous immersion media vs artificial saliva. J. Mater. Sci. Mater. Med..

[26] Atapour M., Standish T.E., Henderson J.D., Wei Z., Dehnavi V., Hedberg Y.S. (2024). Influence of Proteins and Building Direction on the Corrosion and Tribocorrosion of CoCrMo Fabricated by Laser Powder Bed Fusion. ACS Biomater. Sci. Eng..

[27] Aldhohrah T., Yang J., Guo J., Zhang H., Wang Y. (2023). Ion release and biocompatibility of Co-Cr alloy fabricated by selective laser melting from recycled Co-Cr powder: An in vitro study. J. Prosthet. Dent..

[28] (2020). Biological Evaluation of Medical Devices—Part 18: Chemical Characterization of Medical Device Materials within a Risk Management Process.

[29] Saylor D.M., Elder R.M., Duelge K., Ranasinghe Arachchige N.P.R., Simon D.D., Wickramasekara S., Young J.A. (2025). Inter-laboratory study for extraction testing of medical devices. J. Pharm. Biomed. Anal..

[30] Astolfi M.L., Zara L., Antonucci A., Protano C. (2026). Beyond Total Content: Assessing the Bioaccessibility of Metals in Tattoo Inks Using Validated Extraction Media. Anal. Sci. Adv..

[31] Chang H., Tomoda S., Silwood C.J.L., Lynch E., Grootveld M. (2012). ^1^H NMR investigations of the molecular nature of cobalt(II) ions in human saliva. Arch. Biochem. Biophys..

[32] Nikpour S., Hedberg Y.S. (2021). Using chemical speciation modelling to discuss variations in patch test reactions to different aluminium and chromium salts. Contact Dermat..

[33] Richter-Bisson Z.W., Doktor A., Hedberg Y.S. (2023). Serum Albumin Aggregation Facilitated by Cobalt and Chromium Metal Ions. ACS Appl. Bio Mater..

[34] Wei C.X., Burrow M.F., Botelho M.G., Lam H., Leung W.K. (2020). Salivary Protein Adsorption Profile on Titanium and Ceramic Surfaces and the Corresponding Putative Immunological Implications. Int. J. Mol. Sci..

[35] Walter M., Schenkeveld W.D.C., Tomatis M., Schelch K., Peter-Vörösmarty B., Geroldinger G., Gille L., Bruzzoniti M.C., Turci F., Kraemer S.M. (2022). The Potential Contribution of Hexavalent Chromium to the Carcinogenicity of Chrysotile Asbestos. Chem. Res. Toxicol..

[36] Luz M.S., Souza A.L., Haddad A.C.S.S., Tartomano A., Oliveira P.V. (2016). In vitro Cr(VI) speciation in synthetic saliva after releasing from orthodontic brackets using silica-APTES separation and GF AAS determination. Quim. Nova.

[37] Chen H., Davidson T., Singleton S., Garrick M.D., Costa M. (2005). Nickel decreases cellular iron level and converts cytosolic aconitase to iron-regulatory protein 1 in A549 cells. Toxicol. Appl. Pharmacol..

[38] Salnikow K., Donald S.P., Bruick R.K., Zhitkovich A., Phang J.M., Kasprzak K.S. (2004). Depletion of intracellular ascorbate by the carcinogenic metals nickel and cobalt results in the induction of hypoxic stress. J. Biol. Chem..

[39] Sule K., Umbsaar J., Prenner E.J. (2020). Mechanisms of Co, Ni, and Mn toxicity: From exposure and homeostasis to their interactions with and impact on lipids and biomembranes. Biochim. Biophys. Acta Biomembr..

[40] Fels J., Scharner B., Zarbock R., Zavala Guevara I.P., Lee W.-K., Barbier O.C., Thévenod F. (2019). Cadmium Complexed with β2-Microglubulin, Albumin and Lipocalin-2 rather than Metallothionein Cause Megalin:Cubilin Dependent Toxicity of the Renal Proximal Tubule. Int. J. Mol. Sci..

[41] Schmidt M., Raghavan B., Müller V., Vogl T., Fejer G., Tchaptchet S., Keck S., Kalis C., Nielsen P.J., Galanos C. (2010). Crucial role for human Toll-like receptor 4 in the development of contact allergy to nickel. Nat. Immunol..

[42] Schmidt M., Knorz A., Meder K., Goller S., Imdahl F., Rocca Y., Goebeler M., Khoueiry P. (2026). Single Cell Analysis Reveals Dynamic Changes of Distinct Cell Populations in Human Nickel Allergy. Allergy.

[43] Abdoulkader N., Archibald J., Lignereux M., Lehoux E.A., Catelas I. (2025). NLRP3 inflammasome-dependent and -independent interleukin-1β release by macrophages exposed to wear and corrosion products from CoCrMo implants. PLoS ONE.

[44] Mo Y., Nie J., Zhang Y., Zhang Y., Yuan J., Zhang Q. (2025). HDAC6-Mediated NLRP3 Inflammasome Activation Is Involved in Nickel Nanoparticle-Induced Pulmonary Inflammation and Fibrosis. Chem. Res. Toxicol..

[45] Jang E.-Y., Yang S.B., Lee J.-M., Kwack K.H. (2026). Synergistic effects of lipopolysaccharide and titanium ions on monocytic myeloid-derived suppressor cell expansion in gingival tissue: Implications for peri-implant bone health. J. Periodontal Implant Sci..

[46] Kurmangaliyeva S., Baktikulova K., Tkachenko V., Seitkhanova B., Shapambayev N., Rakhimzhanova F., Almagambetova A., Kurmangaliyev K. (2025). An Overview of Hexavalent Chromium-Induced Necroptosis, Pyroptosis, and Ferroptosis. Biol. Trace Elem. Res..

[47] Yuan Y., Hilliard G., Ferguson T., Millhorn D.E. (2003). Cobalt inhibits the interaction between hypoxia-inducible factor-alpha and von Hippel-Lindau protein by direct binding to hypoxia-inducible factor-alpha. J. Biol. Chem..

[48] Afroz S., Amina A., Ansar H., Samreen A., Yusufi F.N.K., Rahman S.A. (2026). Prevalence of metal allergy in patients treated with intraoral prostheses and restorations: A systematic review and meta-analysis. J. Prosthet. Dent..

[49] Ahuja D., Jose N.P., Kamal R., Panduranga V., Nambiar S., Isloor A.M. (2024). In vitro determination of genotoxicity and cytotoxicity induced by stainless steel brackets with and without surface coating in cultures of oral mucosal cells. BMC Oral Health.

[50] Carganico A., Levrini L., Zecca P.A., Mosca A., Deppieri A., Saran S., Giannotta N., Borgese M. (2026). Biocompatibility of orthodontic aligner materials in the digital workflow: A quantitative In Vitro comparison of thermoformed, directly printed, and polyamide-12 systems. J. Dent..

[51] European Parliament and Council of the European Union (2017). Regulation (EU) 2017/745 of the European Parliament and of the Council of 5 April 2017 on medical devices. Off. J. Eur. Union.

[52] (2023). Biological Evaluation of Medical Devices—Part 17: Toxicological Risk Assessment of Medical Device Constituents.

[53] Wu S., Wilsey J., Lewis M., Weng M.-W., Swain Y., Hakimi J., Pereira J., Dai X.S., Rufer E.S., Citrowske S. (2026). Evaluation of the toxicological screening limit and threshold of toxicological concern in medical device toxicological risk assessment for various patient populations. Regul. Toxicol. Pharmacol..

