# Supplementary material for: Release Is Not Dose: Speciation, Bioavailability and Cellular Signalling of Metal Ions from Orthodontic and Prosthetic Alloys in the Oral Environment
Source: Int J Mol Sci. 2026 Oct 20;27(20):9318. doi: 10.3390/ijms27209318 (PMC13705254; doi:10.3390/ijms27209318)
Supplement: Supplementary file 1 [file ijms-27-09318-s001.zip › ijms-4617384-supplementary.pdf]

Supplementary Materials

Release Is Not Dose: Speciation, Bioavailability and Cellular Signalling of Metal Ions from Orthodontic and Prosthetic Alloys in the Oral Environment

Marcin Mikulewicz

Reference numbers follow the main text.

Table S1. Reference values invoked by "below dietary intake" verdicts in the cited release studies, with their units

The table lists the type of reference quantity each source compares its release data with, the numeric value where the source states it, and the unit. None of these quantities is a concentration in an oral fluid; a daily intake (µg/day) or a dose per body weight (µg/kg/day) can be compared with a salivary or extract concentration (µg/L) only by assuming a fluid volume per day, an absorption fraction and a compartment, none of which the comparison states. This is why the verdicts are not plotted in Figure 1.

Ref.	Reference quantity invoked	Numeric value as stated in the source	Unit	Measured quantity it is compared with
[2]	Dietary intake of the same elements from food and drink, normalised to a "standard restored man"	As given in the source (1986 dietary data); not reproduced here	µg/day	In-vitro corrosion-product mass per restored mouth
[3]	Daily dietary intake of nickel; "critical value necessary to induce allergy"	As given in the source; not reproduced here	µg/day; µg (dose)	Ni released per wire into artificial saliva, 1–28 d
[6]	Maximum acceptable concentrations in drinking water; recommended daily doses	As given in the source (national/WHO limits current in 2011); not reproduced here	µg/L (water); µg/day	Ni 573 and Cr 101 µg/L in artificial saliva extract (30 d)
[8]	Tolerable upper intake level (UL) for Ni; derived no-effect level (DNEL) for Ni and Ti; threshold of toxicological concern (TTC) for Ni; EU Nickel Directive release limit	UL 1.0 mg/day (Ni); DNEL 11 µg/kg bw/day (Ni), 350 mg/kg bw/day (Ti); TTC 150 µg/day (Ni); 0.5 µg/cm²/week (skin contact)	mg/day; µg/kg bw/day; µg/day; µg/cm²/week	Ni and Ti release per cm² and per two archwires over 28 d (µg)
[11]	Mean daily dietary intake reported by the World Health Organization (WHO) for Co, Ni, Cr, Ti; "theoretical oral ionic load" via an assumed salivary flow	Co 2.4 (actual 5–40) µg/day; Ni 300–600 µg/day; Cr 50–200 µg/day; Ti 25–30 µg/day; salivary volume assumed 1500 mL/day	µg/day; mL/day	Salivary Co, Ni, Cr, Ti (µg/mL) at 0, 1 and 3 months after debonding

Notes. Values for [8] and [11] are quoted from the open-access full texts; for [2], [3] and [6] the numeric values are stated in the sources and were not re-extracted here, because the point of the table is the unit mismatch, not the magnitude. The analytical detection limits reported in [11] (Ni 11.85 µg/L, Cr 15.74 µg/L, limit of quantification (LOQ) Ni 39.49 µg/L, Cr 53.22 µg/L) exceed the pooled whole-saliva increments of [10] (Table 1 of the main text), which is a further reason for caution in comparing whole-saliva totals across studies.

Table S2. Data harmonisation: conversions, assumptions, sensitivity and the tie-break rule for discordant source values

Conversion	Assumption	Sensitivity	Values tabulated (both where material)	Applies to
ppb → µg/L	Aqueous medium of density 1.00 g/mL	Density 1.03 g/mL changes values by 3%; immaterial on a log axis	Single value	[17]

Conversion	Assumption	Sensitivity	Values tabulated (both where material)	Applies to
μg/g → μg/L (gingival crevicular fluid, GCF)	The authors' published correction [13] applies to all values printed as μg/g in the original article	Factor 10^3; material. Uncorrected reading would place GCF Ni at 19,810 and Cr at 13,760 μg/L	As printed: Ni 3.894 → 19.810 μg/g; Cr 1.978 → 13.760 μg/g. As corrected: same figures in μg/L (used)	[13]
μmol/L → μg/L	Atomic masses Ni 58.69, Cr 52.00, Ti 47.87 g/mol	None beyond element identity	71 μM = 4167 μg/L; 670 μM = 39,322 μg/L (rounded 4170–39,300)	[18]
Increment vs absolute	Pooled mean differences from baseline [10] are increments, not concentrations	Not convertible to absolute values without the baseline of each study	Plotted as increments with open markers	[10]
Per suspension volume vs in situ (biofilm)	Scraping of 3 mm² suspended in 0.5 mL phosphate-buffered saline (PBS) [12]	In-situ concentration depends on assumed biofilm thickness (Section S3)	21.58 μg/L (as published); in-situ estimate in Table S3	[12]
Tie-break for discordant values within one source	Value in the results section tied to the plotted or tabulated data is primary; abstract value reported alongside	[17]: NiTi Ni 210,000 (text) vs >220,000 (abstract), factor 1.05; Ti–Mo Ti 140,000 (text) vs 200,000 (abstract), factor 1.43; SS Ni 500 ppb (abstract, results figure) vs 50 μg/L (another passage), factor 10, unresolved	Both values given in Table 1; primary value plotted in Figure 1	[17]

Section S1. Sequential exposure calculation under stated assumptions

Purpose. Section 7 of the main text states that the evidentiary tiers cannot be converted into one another without assumptions that are presently unavailable. This section makes the conversion anyway, with every assumption stated, so that the size of the gap and its sensitivity to each assumption are visible. The inputs are the measured in-vivo concentrations of Table 1; the comparators are the in-vitro effect regimes of [20]. The result is an order-of-magnitude bracket, not an exposure estimate.

S1.1. Inputs (measured)

Quantity	Value	Molar equivalent	Source
GCF nickel, 6 months, sustained	19.8 μg/L	0.338 μM	[13]
GCF nickel, 30 d peak under fluoridated agents (returned to baseline by 6 months)	101.8 μg/L	1.73 μM	[14]
Biofilm nickel, 9–12 months, median, 0.5 mL suspension of a 3 mm² scraping	21.58 μg/L	0.368 μM	[12]
Whole-saliva nickel increment, ≤10 min / 1 day	+11.5 / +1.38 μg/L	0.196 / 0.0235 μM	[10]

S1.2. Assumptions (stated, not measured)

Assumption	Value(s) used	Basis
A1. Time-weighted epithelial-side concentration equals the measured GCF concentration	19.8 μg/L (sustained) to 101.8 μg/L (peak)	GCF is the fluid in contact with the junctional epithelium and is continuously replaced; at steady state the time-weighted concentration of the fluid is its measured

Assumption	Value(s) used	Basis
		concentration. The 30-d peak was transient, so 101.8 µg/L is an upper bound, not a time-weighted value
A2. Uptake fraction (fraction of fluid-phase Ni that enters epithelial cells)	0.1 to 1.0	Assumed ceiling range; no oral measurement exists. Applied as a multiplier to the fluid concentration to give an "effective supply"
A3. Biofilm thickness over a 3 mm² scraping	50 to 200 µm	Plausible range for mature supragingival plaque adjacent to brackets; not measured in [12]
A4. All nickel recovered in the biofilm suspension was in the scraped biofilm volume	—	Required to back-calculate an in-situ concentration; ignores PBS extraction efficiency and the bound (non-fluid) state of biofilm nickel
A5. Comparator regimes of [20] are transferable across element and tissue	5 and 500 µg/L of Co^{2+} and Cr^{3+} each, primary human bone cells	The only study in the evidence base that defines two concentration regimes and reports the pathway engaged in each; nickel was not tested and the cells were not oral

S1.3. Calculation

Step 1 — crevicular effective supply. Under A1 and A2, the effective supply S = f × C, with C = 19.8–101.8 µg/L and f = 0.1–1.0: S = 2.0–101.8 µg/L (0.034–1.73 µM).

Step 2 — comparison with the systemic regime of [20] (5 µg/L). Ratio C/5 = 4.0 (sustained) to 20.4 (peak); with f = 0.1, S/5 = 0.4 to 2.0. Measured crevicular nickel therefore lies 4- to 20-fold above the concentration at which [20] saw osteoclast effects only, and falls to that regime if only one-tenth is taken up.

Step 3 — comparison with the periprosthetic regime of [20] (500 µg/L). Ratio 500/C = 25 (sustained) to 4.9 (peak); with f = 0.1, 500/S = 250 to 49. Crevicular nickel lies 5- to 25-fold below the regime at which HIF-1α signalling was engaged in osteoblasts, and 50- to 250-fold below it at f = 0.1.

Step 4 — biofilm in-situ concentration. Mass recovered per scraping m = 21.58 µg/L × 0.5 mL = 10.8 ng. Biofilm volume V = 3 mm² × t: for t = 50 µm, V = 0.15 µL; for t = 200 µm, V = 0.60 µL. In-situ concentration m/V = 72 mg/L (t = 50 µm) to 18 mg/L (t = 200 µm), i.e., 18,000–72,000 µg/L or 0.31–1.23 mM. Relative to the sustained GCF value this is 900- to 3600-fold higher, and relative to the ≤10-min whole-saliva increment 1600- to 6300-fold higher, consistent with the "approximately 1000-fold" estimate given by the authors of [12]. The in-situ estimate overlaps (t = 200 µm) or exceeds (t = 50 µm) the ^{1}H NMR titration window of [18] (4170–39,300 µg/L).

Step 5 — comparators that cannot be converted. The NLRP3 activation data of [44] are expressed as 50 µg Nano-Ni per mouse by intratracheal instillation (in vivo) and as a particulate second signal in LPS-primed alveolar macrophages (in vitro); neither is a fluid-phase concentration comparable with GCF, and the particulate form is not the species measured in GCF. The TLR4 activation data of [41] do not define a concentration threshold in a form usable here. No conversion is attempted for either.

Table S3. Summary of fold differences under stated assumptions

Comparison	f = 1.0 (ceiling)	f = 0.1	Assumptions involved
GCF Ni (19.8–101.8 µg/L) vs systemic regime of [20] (5 µg/L)	4.0× to 20× above	0.4× to 2.0× (at or near)	A1, A2, A5
GCF Ni vs periprosthetic regime of [20] (500 µg/L)	25× to 4.9× below	250× to 49× below	A1, A2, A5
Biofilm in situ (18–72 mg/L) vs GCF sustained (19.8 µg/L)	900× to 3600× above	not applicable (bound phase)	A3, A4

Comparison	f = 1.0 (ceiling)	f = 0.1	Assumptions involved
Biofilm in situ vs ^1H NMR titration window (4.17–39.3 mg/L)	within (t = 200 µm) to 1.8× above (t = 50 µm)	not applicable	A3, A4
GCF Ni vs ^1H NMR titration window lower bound (4170 µg/L)	210× to 41× below	2100× to 410× below	A1, A2

S1.4. Sensitivity and interpretation

The crevicular comparison moves by one order of magnitude with the uptake fraction (A2) and by a factor of five with the choice between sustained and peak values (A1); it also crosses elements and tissues (A5), so the bone-cell regimes are a calibration of scale, not a threshold for oral cells. The biofilm estimate moves four-fold with the assumed thickness (A3) and assumes that all recovered nickel was in a fluid phase (A4), which is unlikely for nickel bound to extracellular polymer and bacterial surfaces; the estimate therefore bounds the total, not the bioavailable, biofilm concentration. Two conclusions survive these sensitivities. First, crevicular nickel is not negligible on the scale of the only two-regime in-vitro study available: it sits between the two regimes and reaches the lower one even at a 10% uptake ceiling. Second, the biofilm is the one oral compartment in which total nickel plausibly reaches the concentrations at which salivary speciation has actually been measured, which makes biofilm speciation the most tractable first experiment of Section 8. Neither conclusion is an exposure estimate; each depends on an assumption that no study has measured, and that dependence is what the main text means when it says the tiers cannot be converted without assumptions that are presently unavailable.
